# Reactive Oxygen Species in Planarian Regeneration: An Upstream Necessity for Correct Patterning and Brain Formation

**DOI:** 10.1155/2015/392476

**Published:** 2015-06-09

**Authors:** Nicky Pirotte, An-Sofie Stevens, Susanna Fraguas, Michelle Plusquin, Andromeda Van Roten, Frank Van Belleghem, Rik Paesen, Marcel Ameloot, Francesc Cebrià, Tom Artois, Karen Smeets

**Affiliations:** ^1^Centre for Environmental Sciences, Hasselt University, Agoralaan Building D, 3590 Diepenbeek, Belgium; ^2^Department of Genetics, Faculty of Biology, University of Barcelona and Institute of Biomedicine of the University of Barcelona (IBUB), Avenue Diagonal 643, Edifici Prevosti Planta 1, 08028 Barcelona, Catalunya, Spain; ^3^Faculty of Management, Science and Technology, Open Universiteit, Valkenburgerweg 177, 6419 AT Heerlen, Netherlands; ^4^Biomed, Hasselt University, Agoralaan Building C, 3590 Diepenbeek, Belgium

## Abstract

Recent research highlighted the impact of ROS as upstream regulators of tissue regeneration. We investigated their role and targeted processes during the regeneration of different body structures using the planarian *Schmidtea mediterranea*, an organism capable of regenerating its entire body, including its brain. The amputation of head and tail compartments induces a ROS burst at the wound site independently of the orientation. Inhibition of ROS production by diphenyleneiodonium (DPI) or apocynin (APO) causes regeneration defaults at both the anterior and posterior wound sites, resulting in reduced regeneration sites (blastemas) and improper tissue homeostasis. ROS signaling is necessary for early differentiation and inhibition of the ROS burst results in defects on the regeneration of the nervous system and on the patterning process. Stem cell proliferation was not affected, as indicated by histone H3-P immunostaining, fluorescence-activated cell sorting (FACS), *in situ* hybridization of *smedwi-1*, and transcript levels of proliferation-related genes. We showed for the first time that ROS modulate both anterior and posterior regeneration in a context where regeneration is not limited to certain body structures. Our results indicate that ROS are key players in neuroregeneration through interference with the differentiation and patterning processes.

## 1. Introduction

Reactive oxygen species (ROS) exert a dual role in cells, tissues, and organs [1–3]. On the one hand, diverse pathologies such as neurodegeneration, cardiovascular malfunctioning, and cancer are associated with a disturbed redox balance. On the other hand, the cellular redox signaling also modulates various physiological processes including immunology, development, neurological functioning, wound healing, and angiogenesis [1, 2, 4, 5]. Although many recent studies have extensively investigated this dual role of the redox signature, it remains unclear which redox characteristics direct cells towards a positive or negative outcome.

ROS were recently put forward as early signals in the induction of tissue regeneration [6, 7]. Regeneration is the ability to restore damaged or lost structures without the formation of scar tissue and with complete functional integration in the preexisting body parts. Many organisms can regenerate in early live stages, especially as embryos or larvae, but lose this ability during metamorphosis, puberty, and aging [8]. Some animals maintain excellent regeneration capacities throughout their adult lives, but this varies greatly between species, from invertebrates such as planarians and* Hydra* to vertebrates including* Xenopus* species and axolotl, and between tissues within the same organism. For example, the human liver and skin have good regenerative capacities while neuroregeneration is almost nonexisting in the human body [9–11]. Many studies show that regenerative animals are less vulnerable to aging and are less likely to develop tumors [8, 12–14]. As such, these animal models are crucial to fully understand the regeneration process to overcome or reverse unwanted diseases. Until now, molecular pathways activated during regeneration were extensively studied, but upstream signals remain poorly understood. In 2013, both Love and Gauron described an amputation-induced ROS response during tail regeneration in* Xenopus* and zebrafish, respectively, showing a rapid increase in ROS levels, specifically hydrogen peroxide (H_2_O_2_), at the wound site. Interfering with the ROS burst via NADPH oxidase (NOX) inhibitors impaired regeneration [6, 7, 15]. Accumulating evidence suggests that controlling the intra- and extracellular redox balance holds the answer for the treatment of multiple conditions. de Barros and colleagues (2013) showed that manipulation of ROS signaling (using hypoxic preconditioning) increases the angiogenic capacities of human adipose stroma/stem cells, thereby improving their application in medical therapies [16].

Redox alterations appear crucial in (re-)directing cellular outcomes. In the current study, redox characteristics are linked with stem cell behavior and regeneration, which is essential information that can be used for several (regenerative) applications. A population of pluripotent stem cells, called neoblasts, provides planarians with an unlimited regeneration capacity as they are able to regenerate entire body structures in a matter of days [17–26]. These animals can even regenerate their central nervous system, including the cephalic ganglia, which is a unique ability which only a few species possess [20]. This makes planarians such as* Schmidtea mediterranea* interesting model organisms in regeneration research, especially concerning the process of neuroregeneration [27]. The importance of ROS signaling for correct neuroregeneration was already hypothesized in multiple studies, both* in vivo* and* in vitro *[28–34]. For example, dual oxidas (DUOX)-mediated mediated ROS production is associated with the differentiation of neuronal stem cells [3, 30, 35, 36], whereas in* C. elegans,* a mutation in the peroxidasin (*pxn-2*) gene, which encodes an extracellular peroxidase, results in improved regeneration capacities of mechanosensory axons [37]. Rieger and Sagasti showed that increased hydrogen peroxide levels at the wound site are necessary for peripheral sensory axon regeneration following skin injury in zebrafish larvae [32]. However, these studies still leave some important questions unanswered since they investigated neither the role of redox signaling on regeneration of the central nervous system nor the role of ROS on neuroregeneration in the context of whole body structure regeneration due to limitations of their chosen model system/organism. The remarkable regenerative capacity of planarians makes it is possible to study the importance of ROS production on neuroregeneration as well as on the regeneration of different body structures, thereby comparing both anterior and posterior regeneration. We hypothesize that ROS signaling is necessary not only for posterior regeneration, as indicated by recent studies [6, 7], but also for the regeneration of anterior-situated body structures and more specifically for the formation of a functionally integrated brain. In the current study, we investigated the downstream effects of an impaired ROS burst on both anterior and posterior regeneration in the planarian* S. mediterranea*.

## 2. Materials and Methods

### 2.1. Planarian Cultivation

Asexual strains of the freshwater planarian* S. mediterranea* were maintained in deionized and distilled water containing 1.6 mM NaCl, 1.0 mM CaCl_2_, 1.0 mM MgSO_4_, 0.1 mM MgCl_2_, 0.1 mM KCl, and 1.2 mM NaHCO_3_. The animals were continuously kept in the dark at a temperature of 20°C and were fed once a week with calf liver. Prior to experiments, the worms were starved for at least 7 days [38].

### 2.2. Experimental Design

**Figure 1:**
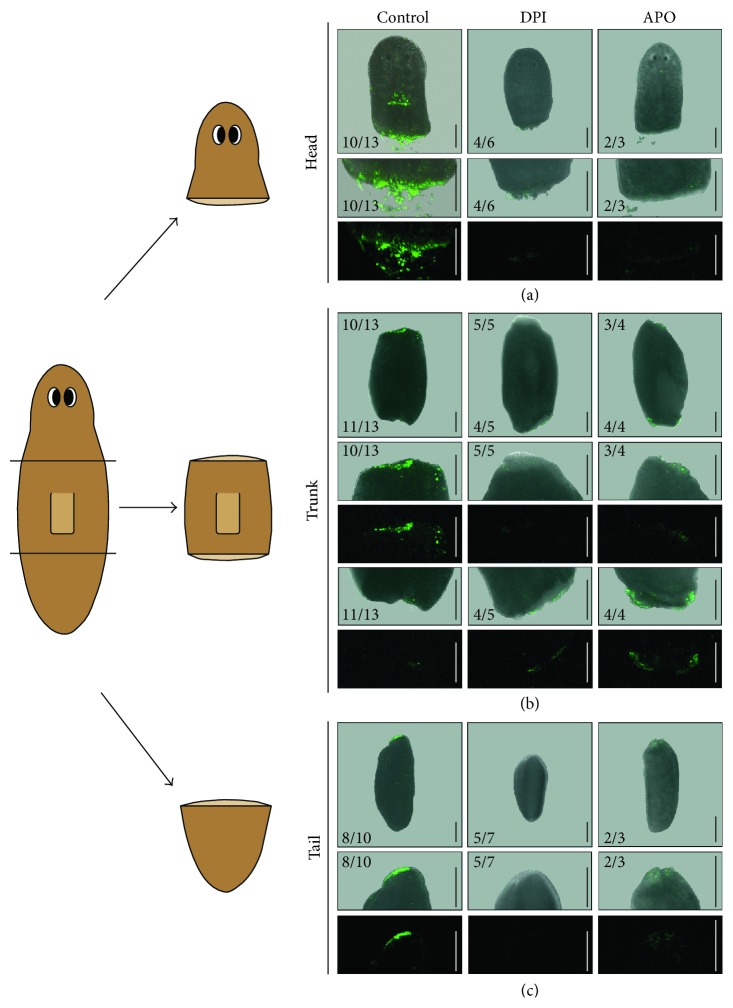
Visualization of ROS levels using carboxy-H_2_DCFDA, 30 minutes post amputation. For each condition a representative image of the entire animal is shown followed by close-ups of both the anterior and posterior wound sites as bright field (upper) and fluorescence (lower) images. (a) ROS levels in regenerating head parts. ROS were produced at the amputation site in control animals (10 out of 13 (10/13) heads displayed fluorescence at the wound site), while ROS levels were visibly diminished in DPI (4/6 displayed diminished fluorescence) and APO-exposed organisms (2/3 displayed diminished fluorescence). (b) ROS levels in regenerating trunks. Amputation-induced ROS were produced at both the anterior and posterior wound site of the control trunk fragment (10/13 trunks displayed fluorescence at the anterior wound sites and 11/13 trunks displayed fluorescence at the posterior wound sites). During DPI and APO exposure, ROS levels were visibly reduced at both amputation sites (DPI: 5/5 displayed diminished fluorescence at the anterior wound sites and 4/5 displayed diminished fluorescence at the posterior wound sites; APO: 3/4 displayed diminished fluorescence at the anterior wound sites and 4/4 displayed diminished fluorescence at the posterior wound sites). A close-up of each wound site is pictured with first the anterior wound site and next the posterior wound site. (c) ROS levels in regenerating tails. ROS are produced at the anterior amputation site (8/10 tails displayed fluorescence at the wound site). DPI and APO exposure reduced ROS levels at the anterior wound site (DPI: 5/7 displayed diminished fluorescence; APO: 2/3 displayed diminished fluorescence). A close-up is shown of each anterior wound site. ROS production in control animals was studied in at least 10 individual fragments. Animals were exposed to 3 *μ*M DPI (*n* ≥ 5) or 400 *μ*M APO (*n* ≥ 3) administered in the cultivation medium. All animals were exposed for at least one hour before the staining procedure. Scale bars total image: 200 *μ*m, scale bars close-ups: 400 *μ*m.

**Figure 2:**
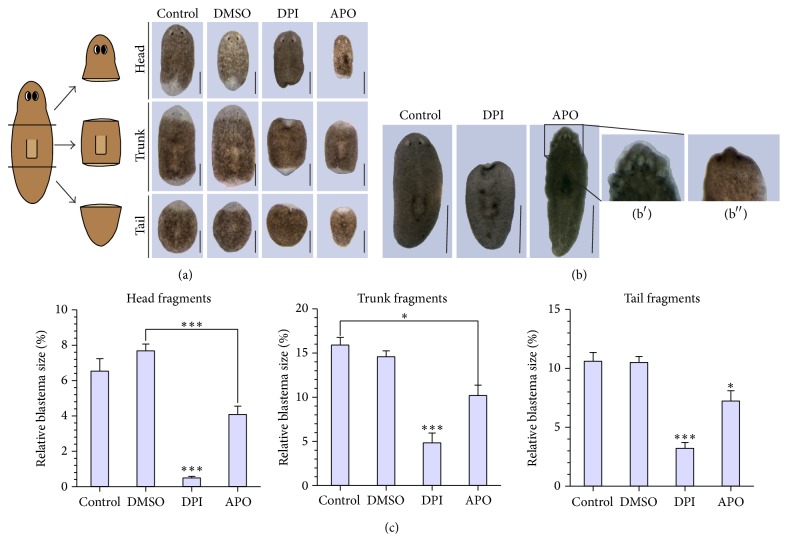
Phenotypical characterization during DPI and APO exposure. (a) The effect of DPI (3 *μ*M) and APO (400 *μ*M) exposure on regenerating head, trunk, and tail fragments after 7 days of regeneration. Animals kept in cultivation medium or a solution of 0.01% DMSO regenerated normally (*n* = 10), while worms exposed to DPI failed to form proper blastemas and photoreceptors (heads: 3/3, trunks: 6/7, tails: 6/6), with a survival rate of 3/10 for the head fragments, 7/10 for trunk fragments, and 6/10 for tail fragments after 7 days of DPI exposure. Additional experiments showed that DPI exposure also diminished blastema formation in a concentration-dependent manner. APO exposure induced similar regeneration defaults, including reduced blastema formation and degeneration of the photoreceptors (3/4 head fragments) or lack of regeneration of the photoreceptors (trunks: 5/5, tails: 5/5), with one head fragment lethality at 7 days post amputation. Scale bar: 500 *μ*m. (b) The effect of DPI and APO exposure on intact organisms. Head regression was observed in all animals exposed to DPI (8/8). APO exposure resulted in the development of lesions, specifically in the anterior region (8/8). Worms were exposed to both inhibitors for 7 days. b′: close-up of an APO-exposed animal. b′′: close-up of a DPI-exposed animal in an early phase of head regression. Scale bar: 1 mm. (c) Presentation of the relative blastema sizes during DPI or APO exposure in comparison to the control animals in regenerating head, trunk, and tail fragments at 7 DPA. The average relative blastema size for the trunk fragments was obtained using the relative sizes of both the anterior and posterior blastemas. (*n* = 10 in both control groups, *n* = 3 DPI-exposed head fragments, *n* = 7 DPI-exposed trunk-fragments, *n* = 6 DPI-exposed tail fragments, and *n* = 5 APO-exposed animals). ^*∗*^ *p* < 0.1; ^*∗∗∗*^ *p* < 0.01. *p* values were obtained via one-way ANOVA analysis. Asterisks show the level of significance. If significant differences were observed between the exposed group and just one of the control groups, a connective line is added between the bars. If the differences are significant in comparison with both control groups, the asterisks are placed above the bar of the exposed group.

**Figure 3:**
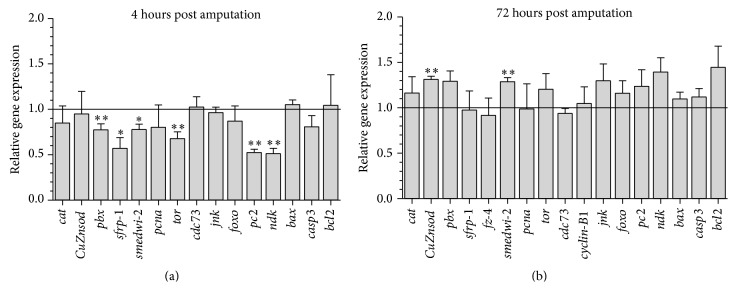
Relative gene expression levels of genes of interest; representing different classes (antioxidative, polarization-related, differentiation-related, proliferation-related, neuronal, and apoptosis-related genes). (a) Gene expression levels of DPI-exposed head fragments relative to the control group (0.01% DMSO) 4 hours post amputation (HPA). (b) Gene expression levels of DPI-exposed head fragments relative to the control group (0.01% DMSO) 72 hours post amputation (HPA). The values indicated in the graphs are the mean ± SEM of minimum 4 biological replicates. There was no effect of DMSO exposure on the expression levels of the measured genes. Significant effects (as compared to the corresponding 0.01% DMSO-exposed control worms): ^*∗*^ *p* < 0.1, ^*∗∗*^ *p* < 0.05. *p* values were obtained via the Kruskal-Wallis test.

Since homologue NOX or DUOX enzymes have not yet been identified in this species, we used two types of ROS inhibitors to test our hypothesis. Diphenyleneiodonium chloride (DPI, Sigma Aldrich, D2926) is a nonspecific flavoprotein inhibitor which interferes with many different electron transporters [3, 39]. Apocynin (APO, 4′-hydroxy-3′-methoxy-acetophenone, Sigma Aldrich, A10809) inhibits the NOX enzymes, acting on the translocation of the cytoplasmic subunits of the enzymes [39, 40]. As such, a maximum reduction of ROS levels during regeneration is ascertained to explore the effects of impaired ROS signaling. After an initial range-finding experiment, we chose to expose the animals to 2 or 3 *μ*M DPI, depending on the type of experiment and time points of interest or to 400 *μ*M APO. Animals were incubated for 1 hour prior to amputation or staining and exposed during regeneration. Experiments were performed on regenerating head, trunk, and tail fragments, unless described otherwise (Figures 1–3). DPI and APO solutions were prepared in 0.01% dimethylsulfoxide (DMSO, Sigma Aldrich, 471267) and a DMSO control group was added to each experiment to investigate possible effects of DMSO exposure. DMSO is a solvent which is regularly used to dissolve hydrophobic compounds. However, in higher concentrations, DMSO is known to influence cell proliferation and have neurotoxic characteristics in* S. mediterranea* [38]. Therefore, we used the lowest concentration of DMSO possible to dissolve both DPI and APO and always added a DMSO-exposed control group.

Buthionine sulfoximine (BSO, Sigma Aldrich, B2515) and oligomycin A (OMA, Sigma Aldrich, 75351) were used to investigate the effects of ROS overproduction during regeneration. A series of concentrations of both substances were used to evaluate the effects of the exposure. Regenerating head, trunk, and tail fragments (*n* = 5) were exposed to either 1 mM and 5 mM BSO or 0.1 *μ*M, 0.5 *μ*M, and 1 *μ*M OMA. Intact animals (*n* = 3) experienced an exposure to 10 mM BSO and 0.05 *μ*M, 0.1 *μ*M, 0.5 *μ*M, 2 *μ*M, and 5 *μ*M OMA. Control groups exposed to either the cultivation medium or 0.05% DMSO were also included during the phenotypical observation. Animals were observed for 4 weeks; every 2 weeks the planarians were fed and the exposure solutions were replaced.

### 2.3. Reactive Oxygen Species (ROS) Detection

The compound 5-(and-6)-carboxy-2′,7′-dichlorodihydrofluorescein diacetate (carboxy-H_2_DCFDA, Image-iT LIVE Green Reactive Oxygen Species Detection Kit, Molecular Probes, Invitrogen, I36007) was used to visualize the production of ROS* in vivo. *We were especially interested in the ROS production at the amputation site of the planarians. Fluorescent carboxy-DCF is produced through ROS oxidation. Planarians were exposed to 3 *μ*M DPI or culture medium for 5 hours prior to staining. Next, the planarians were incubated in carboxy-H_2_DCFDA (25 *μ*M, 1 mL), which was dissolved in their exposure solution, for 1 hour prior to amputation. Amputated animals were again exposed to carboxy-H_2_DCFDA for 15 minutes before immobilization using 0.03% MS222 (ethyl 3-aminobenzoate methanesulfonate, Sigma Aldrich, E10521) and 1% low melting point agarose (Invitrogen). Since the visualization of the ROS production was performed on living organisms, not every image was of sufficient quality due to movement of the animals. Although small differences in fluorescence between anterior and posterior sites may be observed in the selected pictures, no such pattern was detected in the overall observations. Fluorescence detected outside the animal is derived from cells detached from the wound site, as a consequence of the movement. Confocal imaging was performed 30 minutes post amputation using a Zeiss LSM510 META (Carl Zeiss, Jena, Germany) mounted on an Axiovert 200 M (Laser: 488 nm; Filter: BP 500–550 IR; Beam splitters: MBS: HFT 488, DBS1: mirror, DBS2: NFT 490). Quantification of the observed fluorescence was performed using ImageJ (v1.49p, National Institute of Health) and the total pixel intensity at the wound site was normalized to the body size of the animals which was also determined using ImageJ (v1.49p, National Institute of Health).

### 2.4. Immunohistochemistry

Regenerating planarians were fixed and processed as previously described [41]. Primary antibodies used were 3C11 (anti-SYNORF-1, Developmental Studies Hybridoma Bank, dilution 1 : 50), anti-phosphoserine 10 Histone H3 (H3P, Millipore, dilution 1 : 600), and anti-SMEDWI-1 (1 : 1500; Guo et al., 2006 [42]; März et al., 2013 [43]). Secondary antibodies used were Alexa 488-conjugated goat anti-mouse (Molecular Probes, dilution 1 : 400) and Alexa 568-conjugated goat anti-rabbit (Millipore, dilution 1 : 500). To determine the mitotic activity of the stem cells, the total number of mitotic neoblasts was normalized to the body size of the animals, which was determined using ImageJ (v1.49p, National Institute of Health) by measuring the surface of the animals before sampling. Nuclear counterstaining was performed using DAPI (1 *μ*g/mL) overnight at 4°C. The animals were examined with fluorescence microscopy performed with a Nikon Eclipse 80i (Nikon Instruments, Melville, USA) and confocal imaging was performed using a Zeiss LSM510 META (Carl Zeiss, Jena, Germany) mounted on an Axiovert 200 M (Laser: 488 nm; Filter: BP 500–550 IR; Beam splitters: MBS: HFT 488, DBS1: mirror, DBS2: NFT 490), and a Leica TCS-SPE (Leica, Heidelberg, Germany).

### 2.5. Fluorescence-Activated Cell Sorting

Planarian dissociation and cell population analysis were performed as described before [44] with small adjustments. The planarian worms were incubated in 2% L-cysteine HCl (plus 5 M NaOH until pH 7.0) for 2 minutes at room temperature and washed with CMFH (25.6 mM NaH_2_PO_4_·2H_2_O, 142.8 mM NaCl, 102.1 mM KCl, 94.2 mM NaHCO_3_, 0.1% BSA, 0.5% glucose, 15 mM Hepes, and pH 7.2) before being amputated into small pieces (in 250 *μ*L CMFH). Papain solution (30 U/mL CMFH) was added for cell dissociation (1 hour at 26°C). Next DNase I and Trypsin inhibitor were added before cells were filtered (35 *μ*m), collected through centrifugation (350 g, 5 minutes), and resuspended in CMFH plus Calcein AM (1 : 20000; 2 hours of incubation at room temperature, shaking). Ruby was added 30 minutes before flow cytometry analysis using a BD FACSAria II Cell Sorter.

### 2.6. *In Situ* Hybridization

To perform whole mount* in situ* hybridizations, animals were fixed in the same way as with the immunochemistry procedures. Animals were rehydrated through a series of ethanol washes and treated with 20 *μ*g/mL proteinase K (Ambion)/PBST for 5 minutes at 37°C. The proteinase K/PBST was removed with two 5/8 Holtfreter washes and the animals were exposed to 4% paraformaldehyde (PFA)/(5/8) Holtfreter. Tissues were acetylated by incubation in 0.1 M TEA after which 2 × 25 *μ*L of acetic anhydride was added. The animals were washed with PBS before being incubated in prehybridization buffer for at least 1 hour at 56°C. Hybridization was carried out for at least 16 hours at 56°C in hybridization buffer. Afterwards, samples were washed through a series of posthybridization buffers and buffer I (0.01 M maleic acid, 0.15 M NaCl, 0.15 M NaOH, and pH 7.5) and next blocked in Buffer II (Buffer I with 1% Blocking Solution). Samples were incubated at RT for 3 hours in 1 : 2000 anti-DIG/Buffer II. The antibody was removed by washing with Buffer I. Colour development was performed by incubation of the samples in 20 *μ*L/mL NBT/BCIP at RT. When the colour reaction was complete, the animals were washed with PBS and fixed in 4% PFA/PBS. A series of ethanol washes was performed to optimize the colour development [45, 46]. The RNA* in situ* probes were synthesized using the DIG RNA labeling kit (Sp6/T7, Roche) following manufacturer's instructions (*smedwi-1*, forward primer: GTGACGCAGAGAAACGGAAG, reverse primer: TTGGATTAGCCCCATCTTTG;* smed-gpas*, forward primer: GCGGAAAAAGCCATGAAAG, reverse primer: CGACTTCGTAGCACATCCTG [23];* smed-sfrp-1*, forward primer: AATGTACGGATTTGACTGG, reverse primer: CGATTGTTGGGTTTGACT [47];* smed-notum*, forward primer: CGAGTGATTTGTGGTCTGG, reverse primer: CGTGGAGTCGTTGATTGTTG [48];* smed-fz-4*, forward primer: TGTTTGGGGCGATTTTGG, reverse primer: GGGTCGGTTCTTCTTCTTT [48]. Fluorescent* in situ* hybridization was performed using the protocol published by King and Newmark [49]. The number of stained cells in the prepharyngeal area was determined using the Cell Counter plugin of ImageJ and the number of positive cells was normalized to the surface area of the prepharyngeal region. Bright field images were digitized on a Nikon SMZ800 (Nikon Instruments, Melville, USA) using a DFK 41AF02 camera (The ImaginSource, Bremen, Germany) or using a ProgRes C3 camera (Jenoptik, Jena, Germany). Confocal imaging was performed using a Zeiss LSM510 META (Carl Zeiss, Jena, Germany) mounted on an Axiovert 200 M (Laser: 488 nm; Filter: BP 500–550 IR; Beam splitters: MBS: HFT 488, DBS1: mirror, DBS2: NFT 490), and a Leica TCS-SPE (Leica, Heidelberg, Germany).

### 2.7. qPCR

RNA or DNA was isolated using a phenol-chloroform extraction procedure [50] and was precipitated with Na-acetate and 70% ethanol. Nucleotide concentrations were assessed on the NanoDrop ND-1000 spectrophotometer (NanoDrop Technologies). For RNA samples, genomic DNA was removed with the Turbo DNA free kit (Ambion). cDNA was synthesized using the Superscript III First-Strand Synthesis SuperMix for qRT-PCR (Invitrogen) following manufacturer instructions. DNA samples were treated with RNase A (Promega) to delete possible RNA contaminations.

Real-time PCR was performed in an optical 96-well plate using the ABI PRISM 7900 (Applied Biosystems) under universal cycling conditions. SYBR Green (Applied Biosystems) chemistry-based real-time PCR was carried out [51]. The selection of potential reference genes was based on Plusquin et al. (2012) [52], after which the most stable reference genes during DPI exposure were determined by Normfinder and geNorm analysis. Gene expression analyses were performed with MIQE guidelines taken into account [53]. Details of the procedure are given in Supplementary Table 2 (see Supplementary Material available online at http://dx.doi.org/10.1155/2015/392476).

We measured the amount of mitochondrial DNA (mtDNA) compared to the amount of nuclear DNA (nDNA) between control (medium and 0.01% DMSO) and DPI-exposed (2 *μ*M) head fragments. Genes used to measure mtDNA were* cyb* (cytochrome b, forward primer: TGTCTCTTTGGGGAGCTACTG, reverse primer: CCACCTCACAACCACTCAAC) and* co1* (cytochrome oxidase subunit 1, forward primer: CTGTTATGATTGGAGGATTTGG, reverse primer: CATATTATTAGCACGAGGAAAGG) while the nDNA was measured using* alas1* (aminolevulinate, delta-synthase 1, forward primer: ATACGCGAAACGATCCAAAC, reverse primer: AGTCTATCCACCCAGCCAGA),* cat* (catalase, forward primer: TTCCTCAGAAAACCGCATAGA, reverse primer: TTTTCATTTCTCCGCCAAC), and* smed-NB.21.11e* (novel gene, forward primer: GTCTCCCGCCAAATCAAGTA, TTTCATGCAATCTGCTTTCG). Analyses were performed in a similar way as the qPCR experiments.

### 2.8. Statistics

After normality was checked, groups were statistically compared using either one-way ANOVA analyses or the Kruskal-Wallis test. *p* values less than 0.1 were considered significant. The statistical analyses were performed using R 3.0.2.

## 3. Results and Discussion

Recent studies show that ROS are able to modulate the process of regeneration. Before these findings can be extrapolated to regenerative and therapeutic applications, information must be gathered on the specific role and downstream targets of ROS. Our results supplement previous findings as we investigated the effects of an impaired ROS burst on processes such as proliferation, differentiation, and patterning. We used an organism with unlimited regenerative capacities, the planarian* Schmidtea mediterranea*, and compared the effects of a decreased ROS burst on both anterior and posterior regeneration [17–26].

### 3.1. Reactive Oxygen Species Are Produced during Wound Healing

To evaluate the ROS burst during both anterior and posterior regeneration, organisms were amputated along two planes, one in front and one behind their pharynx, creating three individual organisms (head, trunk, and tail) as illustrated in the figures. We used carboxy-H_2_DCFDA to visualize nonspecific ROS levels, a ROS detection method which has been applied in various experimental conditions, including in regeneration contexts [6, 54]. The observed ROS production at the wound sites (Figure 1) correlated with the results shown in zebrafish and* Xenopus* studies [6, 7]: a rapid ROS production at the wound sites within minutes after amputation (Figure 1). The orientation of the wound did not influence the intensity of the amputation-induced ROS burst, but the ROS burst at both wound sites of the trunk fragments was less intense compared to the ROS-induced fluorescence at the wound sites of the head and tail fragments (Figure 1; supplementary Figure 1(A)). The signal was diminished in all body fragments after exposure to two different types of inhibitors, diphenyleneiodonium (DPI, a nonspecific flavoprotein inhibitor) and apocynin (APO, an inhibitor of NOX-like enzymes) (Figure 1). In Figure 1, a representative fragment of each group is shown. The observed signals are not the result of nonspecific autofluorescence, since no signal was detected in the unstained control fragments (supplementary Figure 1(B)).

### 3.2. Amputation-Induced ROS Are Needed for Proper Blastema Formation

The amputation-induced ROS burst is crucial for successful regeneration (Figure 2). Both ROS inhibitors DPI and APO noticeably reduced fluorescent signaling of ROS at all the different wound sites (Figures 1(a)–1(c), supplementary Figure 1(A)) causing improper regeneration of all fragments, similar to the observed tail regeneration defaults in* Xenopus* and zebrafish studies [6, 7]. Although no differences in ROS production were observed between anterior and posterior wound site, ROS inhibition was most effective in the head fragments (supplementary Figure 1(A)). This correlates with the observed differences in vulnerability between the different body fragments. Head fragments were more susceptible (especially to DPI exposure) in comparison to trunks and tails resulting in higher mortality rates and more significant effects on the regeneration capacity (Figure 2). In the regenerating head fragments, we investigated the effects of the DPI exposure on the redox balance via gene expression analyses and noticed that not only prooxidant levels, but also antioxidant levels were affected as shown by the upregulation of the antioxidative enzyme* CuZnSOD* at 72 HPA (hours post amputation, *p* = 0.016, Figure 3, supplementary Table 1).

Regeneration defects due to the diminished ROS burst include reduced blastema sizes, lack of photoreceptor formation, and loss of preexisting photoreceptors (Figures 2(a) and 2(c)), phenotypes which were observed in each body fragment exposed during regeneration (total number of DPI-exposed fragments over various experiments = 293). DPI-induced defects on blastema size as well as lethality were more profound in comparison with the observed defects in APO-exposed animals. The more severe defects caused by DPI could be explained by the nonspecificity of the compound (Figure 1). While APO specifically blocks NOX-like enzymes, DPI can also disturb other sources of ROS production, like the mitochondrial respiratory chain [55, 56]. Therefore, we investigated whether DPI exerted an effect on regeneration via interference with the mitochondrial metabolism, which is linked with mitochondrial abundance [57]. No effect of DPI exposure was detected on the mitochondrial abundance (supplementary Figure 2); therefore it seems that the number of mitochondria and their metabolic functioning is not influenced by the inhibition of the amputation-induced ROS burst.

Although a decreased ROS level at the wound site resulted in a failure of regeneration, a disturbance of the redox balance in favor of ROS production did not affect the regenerative capacity. Exposure to two types of ROS-promoting compounds, buthionine sulfoximine (BSO, inhibitor of gamma-glutamylcystein synthetase, thereby inhibiting glutathione synthesis) and oligomycin A (OMA, inhibitor of ATP synthase) did not cause any noticeable regeneration defects or phenotypical abnormalities in intact animals, although an overproduction of ROS at the wound site was observed (supplementary Figure 3). These data demonstrate that although regenerating organisms need ROS signaling for correct regeneration, they are able to cope with increased ROS levels without any regeneration defaults, probably through the activation of the antioxidative systems and DNA repair machineries in the stem cells [58].

In intact organisms, similar effects were detected. Also in these animals, the anterior region was most vulnerable to redox manipulations (i.e., less ROS production). A DPI exposure resulted in a regression of the heads and animals exposed to APO developed lesions at their head region after approximately 7 days of exposure (Figure 2(b)). Long-term exposure (≥7 days) to both inhibitors eventually resulted in death of the animals. The increased vulnerability of the head regions and fragments might be the result of ROS production in the brain as we observed ROS-induced fluorescence in neuronal-like structures (supplementary Figure 1(C)). Therefore, we investigated the effects of an inhibition of neuronal-derived ROS by diminishing the expression of monoamine oxidase (*mao*), an enzyme that produces ROS as a side product of neurotransmitter degradation [59, 60], through RNA interference (RNAi). Although the* mao* knockdown did not result in an impaired regeneration, it was lethal for regenerating head pieces and resulted in the formation of lesions in the head region of intact animals and in regenerating trunk and tail pieces (supplementary Figure 4), comparable to the phenotypic effects caused by APO exposure. The dissimilar effects of* mao(RNAi)* and DPI/APO exposure on blastema formation might be due to the restricted localization of the ROS production in the neurons or the limited effects of the RNAi treatment on neuronal maintenance.

Overall, since DPI induced the strongest ROS decrease resulting in the most severe phenotypical effects in both regenerating and intact organisms, we continued with DPI in the following experiments to further investigate the impact of an impaired ROS burst on key processes involved in regeneration (proliferation, differentiation, and patterning).

### 3.3. Reduced ROS Levels Do Not Affect Wound Closure

**Figure 4:**
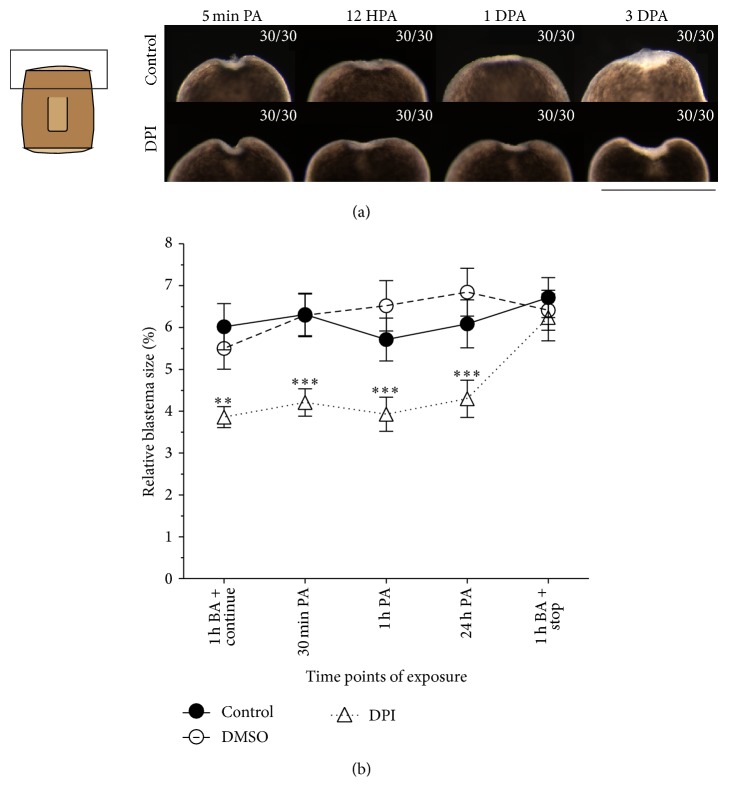
The effects and time points of DPI exposure on (early) regeneration. (a) DPI exposure does not inhibit wound closure or early blastema formation. Contractions of the muscles at the wound site are observed in both control and DPI-exposed animals directly after amputation. At early time points of regeneration (12 HPA and 1 DPA) reepithelialization and early blastema formation occured normally in DPI exposed organisms while at later time points (3 DPA) the effect of a diminished ROS production on blastema size was clearly noticeable. Scale bar: 500 *μ*m. (b) Inhibition of the ROS burst exerted an effect on blastema formation at a later time point of regeneration. A similar reduction of blastema size was observed in animals exposed to DPI at 30 minutes, 1 hour, or 24 hours post amputation in comparison to the inhibition before amputation (*n* ≥ 4). When animals were exposed to DPI 1 hour before amputation (BA) but were repositioned in culture medium during regeneration, no reduction in blastema size was observed. Relative blastema sizes were determined at 7 days post amputation. ^*∗∗*^ *p* < 0.05. ^*∗∗∗*^ *p* < 0.01. *p* values were obtained via one-way ANOVA analyses.

Although the inhibited ROS production clearly affected blastema formation, DPI exposure did not influence wound closure or early phases of regeneration (Figure 4(a)). To investigate the time point at which ROS signaling is necessary for correct regeneration to proceed, we exposed regenerating planarians at different time points during regeneration, either before or after amputation. Exposure to the inhibitor 1 hour before amputation or 30 minutes, 1 hour, and even 24 hours post amputation all resulted in a similar blastema size reduction (Figure 4(b)). When we exposed the animals before amputation (BA) but placed them in culture medium in absence of the inhibitor during the regeneration process, the exposed animals were able to form a normal blastema (Figure 4(b)). These data suggest that although the ROS burst is present within minutes after the amputation, the necessity of ROS signaling for successful regeneration happens at a later time point (later than 24 HPA).

### 3.4. Reduced ROS Levels Do Not Affect Stem Cell Proliferation

**Figure 5:**
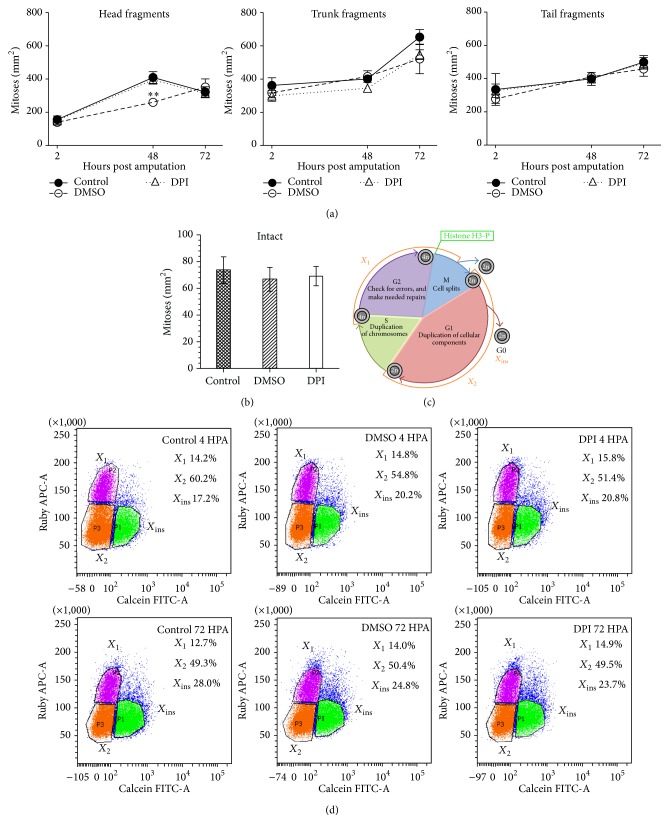
DPI exposure does not affect stem cell proliferation. (a) Cell proliferation in DPI-exposed (2 *μ*M) head, trunk, and tail fragments at 4, 48, and 72 hours post amputation. No significant effects of DPI exposure on cell proliferation were observed (*n* ≥ 3). (b) DPI exposure did not affect stem cell proliferation in intact animals. 7 days of DPI exposure before immunostaining (*n* ≥ 5). (c) Schematic figure of the cell cycle and visualization of the different stem cell populations. (d) FACS data of the three stem cell populations (*X*_1_, *X*_2_, and *X*_ins_) during DPI exposure in comparison with control animals at 4 HPA and 72 HPA. Each sample at 4 HPA existed of 6 regenerating head fragments, while measurements at 72 HPA were performed with 4 animals/sample. No effects of the DPI exposure were observed on any of the different neoblast populations or on their distributions.

Stem cells (neoblasts) are the underlying force of successful regeneration [17, 19, 24, 25]. Despite drastic regeneration defects caused by a diminished ROS production, DPI did not affect the regular stem cell proliferation peaks at 4 hours and 72 hours post amputation. Neither the number of cells in the G2/M phase at 4 HPA, 48 HPA, or 72 HPA (Figure 5(a)), nor the different neoblast populations (*X*_1_: proliferating neoblasts in G2/M phase; *X*_2_: nonproliferating neoblasts in G0/G1 phase; *X*_ins_: differentiating cells) were altered during DPI exposure in regenerating fragments (Figures 5(c) and 5(d)) [61]. Gene expression analyses of the neoblast-specific proliferation marker* pcna* and cell cycle regulating genes* cdc73* and* cyclinB-1* confirm the lack of effects on stem cell proliferation (Figure 3, supplementary Table 1). However, gene expression of* tor* (target of rapamycin) was downregulated during DPI exposure in regenerating head fragments at 4 HPA (*p* = 0.032) (Figure 3, supplementary Table 1). TOR is a widely conserved and important regulator of cell growth and proliferation, controlling the progression of the G1 phase to the S phase of the cell cycle [62–64]. Mammalian studies have reported that ROS (H_2_O_2_) activate TOR and its target S6 kinase [65]. Moreover, TOR is not only activated by increased ROS levels, TOR itself also regulates the production of ROS [65]. Literature shows that, similar to DPI-exposed phenotypes,* tor(RNAi)* fragments are unable to form recognizable blastemas and regenerate the nervous system structures (e.g., brain, visual neurons) within preexisting tissues which do not require the formation of these structures [62–64]. Intact* tor(RNAi)* treated animals develop lesions, comparable to the APO phenotype, but these lesions were present in the postpharyngeal region instead of the head region [62, 64]. This suggests that the cell cycle might not be completely unaffected by an impaired ROS production, as was also shown by the decrease in proliferation which was observed in regenerating tails and fins of* Xenopus* and zebrafish, respectively, after ROS reduction [6, 7]. On the other hand, we must keep in mind that proliferative responses during regeneration in* Xenopus* species and zebrafish were measured on mitotic, dedifferentiated epidermal cells, while regeneration in* S. mediterranea* is the result of proliferation of pluripotent stem cells.

Similar to the regenerating parts, stem cell proliferation was also unaffected by a diminished ROS production in intact animals (Figure 5(b)). The number of mitotic cells after 7 days of DPI exposure did not differ from the number of mitoses in control animals. Since regeneration defaults and homeostatic imbalances caused by a diminished ROS production are not induced by a reduced proliferation, ROS signaling must affect other processes involved in regeneration.

### 3.5. An Inhibition of ROS Production Affects Early Neoblast Differentiation

**Figure 6:**
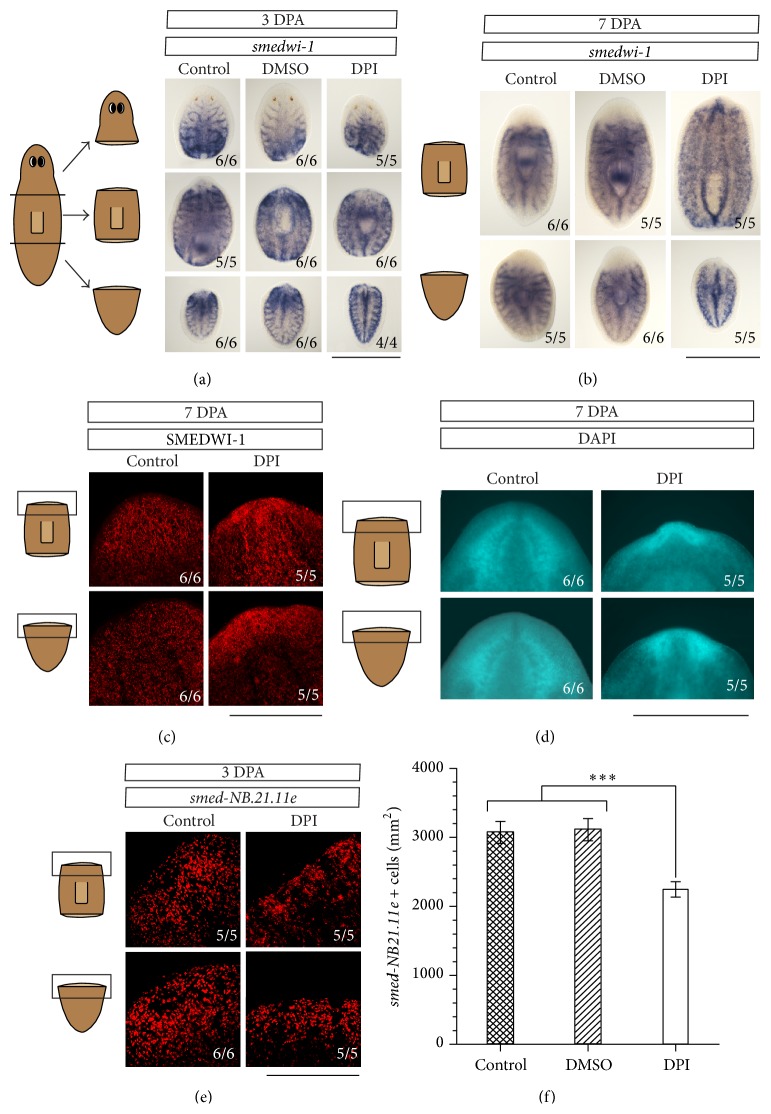
Effects of DPI exposure on neoblast differentiation. (a)* Smed-wi1* expression at 3 days post amputation (3 DPA) in head, trunk, and tail fragments. Exposure to DPI (3 *μ*M) did not affect the expression (pattern) of the* smedwi-1* gene (*n* ≥ 4). Scale bar: 1 mm. (b)* Smedwi-1* expression at 7 days post amputation (7 DPA) in head, trunk, and tail fragments. Exposure to DPI (3 *μ*M) did not affect the expression (pattern) of the* smedwi-1* gene (*n* ≥ 5). Scale bar: 1 mm. (c) An increase of SMEDWI-1 positive cells was observed at the wound sites of trunk and tail fragments after exposure to DPI (3 *μ*M) in comparison to the control fragments at 7 days post amputation (7 DPA) (*n* ≥ 5). Scale bar: 500 *μ*m. (d) An increase in cell density was noticeable at the wound site of the DPI-exposed animals after 7 days of regeneration (7 DPA) in comparison to the control animals (*n* ≥ 5). Scale bar: 500 *μ*m. (e) A decrease of early neoblast progeny, marked by the expression of* smed-NB21.11e*, was observed in DPI-exposed (3 *μ*M) trunk and tail fragments (*n* ≥ 5). Scale bar: 500 *μ*m. (f) Quantification of the number of* smed-NB.21.11e*-positive cells. The number of cells was counted in the prepharyngeal area of the regenerating trunk and tail fragments. A significant decrease was observed in the DPI-exposed trunk and tail fragments in comparison to the trunks and tails of the control groups. ^*∗∗∗*^ *p* < 0.01. *p* values were obtained via one-way ANOVA analysis.

During regeneration, stem cells not only need to proliferate, but also need to differentiate to regrow the missing tissues. Various redox-modulated signaling pathways, such as the MAPK cascades, regulate this differentiation process [66]. Since stem cell proliferation was not affected after inhibition of the ROS production, the effect of DPI exposure on differentiation was investigated. Both the expression of* smedwi-1* and the presence of the SMEDWI-1 protein were studied in control and DPI-exposed animals. During normal regeneration,* smedwi-1*-expressing neoblasts migrate to the wound site where they will differentiate to form the blastema. As the neoblasts differentiate, they no longer express* smedwi-1*; however, the protein will remain present in the early neoblast progeny and will eventually degrade in the differentiated cells [66, 67]. Therefore,* smedwi-1* expression is observed throughout the mesenchyme of the animal and this gene is not highly expressed within the blastema. After DPI exposure,* smedwi-1* expression was not affected at an early (3 DPA, Figure 6(a)) or a later (7 DPA, Figure 6(b)) time point of regeneration.* Smedwi-1* was expressed in similar quantities and patterns in all the regenerating organisms. These data confirm that the defects on blastema formation do not result from depletion of the stem cell population. When the SMEDWI-1 protein was observed, the SMEDWI-1 positive cells were normally distributed within the anterior blastema of the control trunk and tail fragments, indicating that the blastemas consist of early neoblast progeny and differentiated cells. However, in DPI-exposed animals, these SMEDWI-1-positive cells were more abundant in comparison with the control animals and accumulated at the wound sites (Figure 6(c)), suggesting that those neoblasts in the DPI-exposed animals fail to fully differentiate. This increased cell density was also observed in the blastemas of DPI-exposed organisms with the DAPI staining, which visualizes the nuclei of the cells (Figure 6(d)). These data suggest that the SMEDWI-1-positive cells are unable to proceed to a differentiated state which confirms that the phenotypical defects observed after exposure to the ROS inhibitor do not result from any effects on neoblast proliferation or survival but rather result from an effect on stem cell dynamics. To clarify the effects of DPI exposure on early neoblast differentiation, the number of cells expressing* smed-NB21.11e*, a marker for early neoblast postmitotic progeny [68], was determined in the prepharyngeal area at 3 DPA using fluorescent* in situ* hybridization. The number of early neoblast progeny cells significantly decreased in both DPI-exposed trunk and tail parts in comparison with the control animals (27-28% less* smed-NB21.11e*-positive cells in DPI-exposed trunk and tail fragments in comparison with control (*p* = 0.0014) and DMSO-exposed animals (*p* = 0.0008), Figures 6(e) and 6(f)) confirming that ROS signaling is necessary for proper early neoblast differentiation rather than neoblast proliferation or maintenance. Gene expression analyses reinforce this hypothesis since expression levels of* smedwi-2* were significantly downregulated at 4 HPA (*p* = 0.056) and upregulated at 72 HPA (*p* = 0.016) in DPI-exposed regenerating head fragments. SMEDWI-2 is an enzyme which is needed for the production of neoblast progeny capable of replacing aged differentiated cells during homeostasis and missing tissues during regeneration [67].* Smedwi-2(RNAi)* planarians display defects comparable to the DPI- and APO-exposed phenotypes, including regression of the tissue anterior to the photoreceptors and incapability of regeneration [67]. Also,* smedwi-2(RNAi)* did not affect the normal proliferative wounding response of the neoblasts, since stem cells were able to proliferate and migrate to the wound site [67].

### 3.6. An Inhibition of ROS Production Disturbs Neuroregeneration

**Figure 7:**
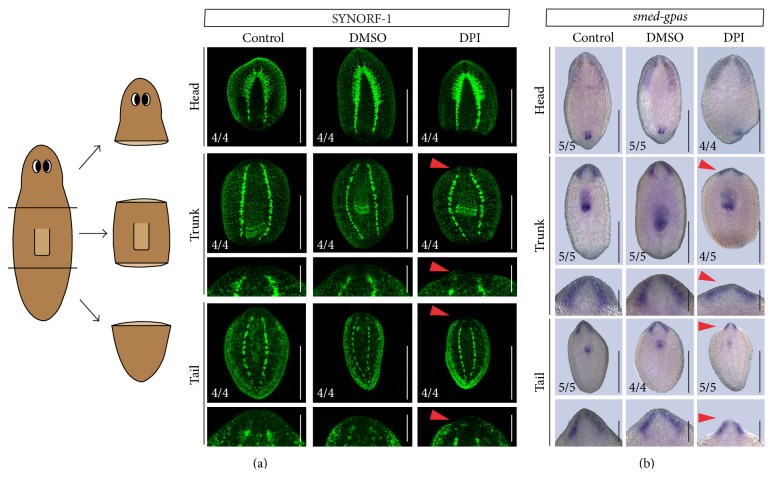
DPI exposure impairs proper neuroregeneration. (a) Regenerating head, trunk, and tail fragments exposed to either control medium, 0.01% DMSO, or DPI (2 *μ*M) during a regeneration period of 5 days were stained using anti-SYNORF-1, visualizing the central nervous system (CNS). In the DPI-exposed trunks (4/4) and tails (4/4), the amputated brain failed to regenerate properly, indicated by red arrow heads. (*n* = 4). Close-ups of the blastemas were presented below the respective figure for the regenerating trunk and tail fragments. Scale bars: 1 mm. Scale bars close-ups: 500 *μ*m. (b)* Gpas* expression in 7-days-regenerating head, trunk, and tail fragments visualized using* in situ* hybridization. Reduction of the cephalic ganglia in DPI-exposed trunks (4/5) and tails (5/5) was observed, as well as improper formation of the regenerating pharynx in regenerating head pieces (4/4). Close-ups of the blastemas are presented below the respective picture of regenerating trunk and tail fragments. Scale bars: 1 mm. Scale bars close-ups: 500 *μ*m.

To characterize the effects of ROS inhibition on differentiation, we investigated the regeneration of the central nervous system since we observed a decrease in the size of the brain in the DPI-exposed animals visualized by the DAPI staining (Figure 6(d)). Although the importance of ROS signaling for proper neuroregeneration has been identified before, we show for the first time their involvement in the restoration of the central nervous system (CNS) and more specifically in the regeneration of the brain. DPI-exposed trunk and tail fragments had reduced cephalic ganglia in comparison with the control animals, which was visualized by both immunohistochemistry using an anti-SYNORF-1 antibody and* in situ* hybridization of the* smed-gpas* gene (Figures 7(a) and 7(b)). In accordance with these data, the transcript levels of prohormone convertase 2 (*pc2*), a neurotransmitter convertor [69], and nou-darake (*ndk*), a* fgfr*-related gene which restricts brain development to the head region [70], were significantly reduced during DPI exposure (*pc2*: *p* = 0.016;* ndk*: *p* = 0.016), which showed that correct regeneration of the (central) nervous system relies on ROS signaling (Figure 3, supplementary Table 1).

**Figure 8:**
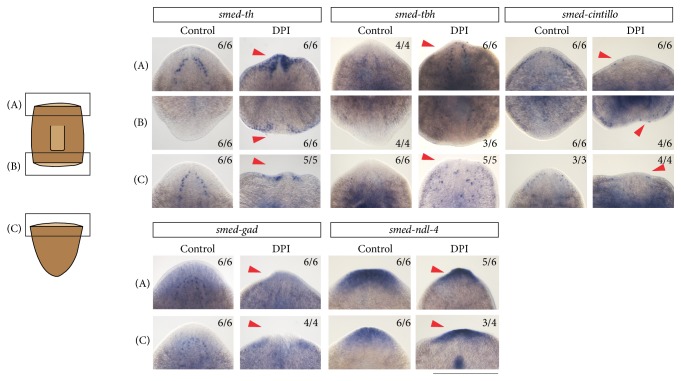
The expression of specific neuronal markers after DPI exposure at 7 days post amputation (7 DPA). The* smed-th* and* smed-tbh* expressing cells noticeably increased at the anterior blastemas of DPI-exposed trunk (*n* = 6/6) and tail fragments (*n* = 5/5) in comparison to control organisms, while* smed-cintillo* (trunks: *n* = 6/6; tails: *n* = 4/4) and* smed-gad *(trunks: *n* = 6/6; tails: *n* = 4/4) expressing cells were clearly reduced at the anterior sites after DPI exposure (3 *μ*M). At the posterior wound sites of the DPI-exposed trunks, expression of* smed-th* (*n* = 6/6),* smed-tbh* (*n* = 3/6) and* smed-cintillo* (*n* = 4/6) was observed which was absent in the control animals. Expression of the anterior marker* smed-ndl-4* was noticeably increased in DPI-exposed trunks (*n* = 5/6) and tails (*n* = 3/4). Differences in the expression of these genes are indicated in the figure by red arrow heads. Scale bar: 500 *μ*m.

To obtain more information concerning the effect of a diminished ROS burst on the regeneration of the cephalic ganglia, we investigated the formation of various types of neurons after DPI exposure by using additional neuronal and anterior markers (Figure 8). After 7 days of regeneration the expression of* smed-cintillo* (mechanosensory neurons, [71]) and* smed-gad* (GABAergic neurons, [72]) was clearly reduced at the anterior site after DPI exposure, which might indicate that there are problems with the differentiation of these neuronal cells. Interestingly, the expression of* smed-th* (dopaminergic neurons, [73, 74]),* smed-tbh* (octopaminergic neurons, [22, 75]), and* smed-ndl-4* (a FGF receptor-like protein of the* nou-darake* family [70, 76]) seemed to be upregulated at the anterior blastema in the DPI-exposed trunks and tails in comparison with the control animals. However, the observed accumulation of cells expressing these neuronal genes might be the result of the reduced blastema and the mispatterning of the brain (Figure 7). It is not clear whether there are actually more* smed-th-*,* smed-tbh*-, and* smed-ndl-4*-expressing cells in the blastema or whether they are mispatterned without an additional number of cells. On the other hand, we also observed ectopic expression of* smed-th* and* smed-cintillo* in the posterior blastemas in the DPI-exposed trunk fragments while these neurons are normally absent at the posterior wound site. The formation of these neuronal cells in the posterior region suggests that diminished ROS levels result in identity issues of the differentiating neoblasts and that ROS signaling is necessary to not only initiate but also coordinate the differentiation process.

### 3.7. An Inhibition of ROS Production Affects Proper Patterning and Polarity

**Figure 9:**
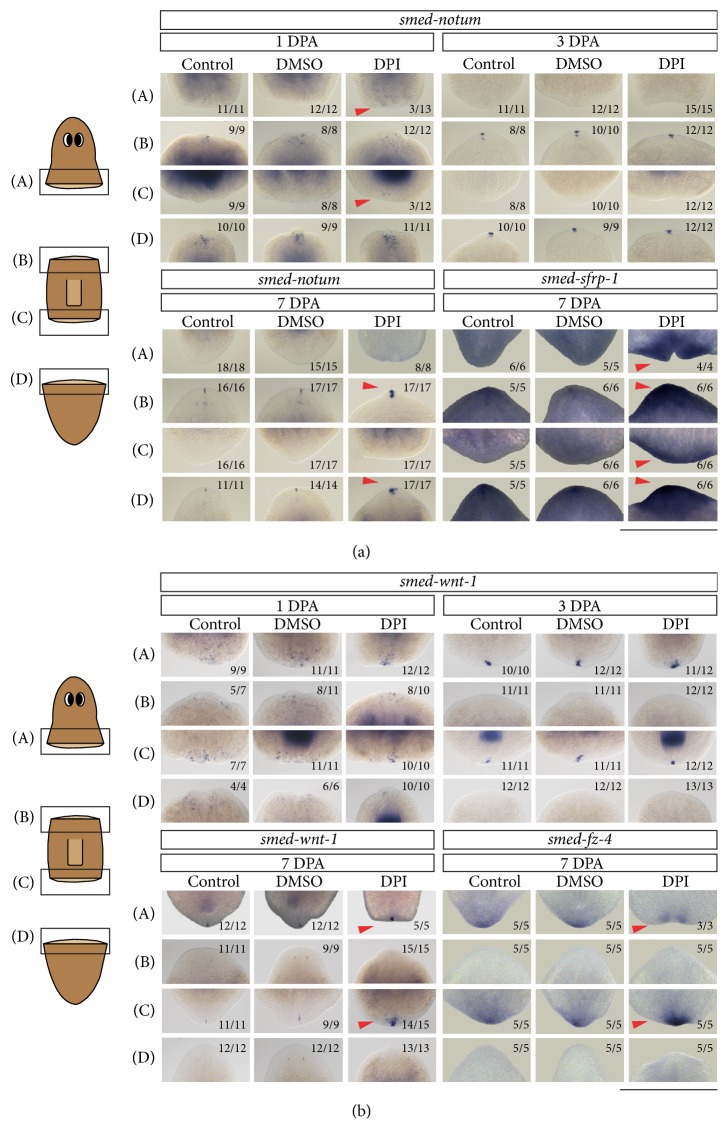
*In situ* hybridizations of anterior (*smed-notum* and* smed-sfrp-1*) and posterior (*smed-wnt-1* and* smed*-*fz-4*) markers at different time points during regeneration after DPI exposure. (a) The expression of anterior markers* smed-notum* and* smed-sfrp-1* in regenerating head, trunk, and tail fragments at different time points during regeneration. Posterior expression of* smed-notum* was observed after DPI exposure (3 *μ*M) at 1 DPA in both head (*n* = 3/5) and trunk fragments (*n* = 3/6). At 7 DPA, an increased expression of both* smed-notum* and* smed-sfrp-1* was observed in anterior blastemas of the DPI-exposed trunk and tail fragments in comparison to the control animals. Differences in gene expression are indicated with red arrow heads. Scale bar: 500 *μ*m. (b) The expression of posterior markers* smed-wnt-1* and* smed-fz-4* in regenerating head, trunk, and tail fragments at different time points during regeneration. Increased expressions of* smed-wnt-1* and* smed-fz-4* were observed at 7 DPA in DPI-exposed trunk and tail fragments (3 *μ*M) in comparison to control animals. Differences in gene expression are indicated with red arrow heads. Scale bar: 500 *μ*m.

Based on the diminished cephalic ganglia, posterior expression of neuronal cells, and seemingly increased expression of the anterior marker* smed-ndl-4*, we investigated the effect of an inhibited ROS production on polarization and patterning.* In situ* hybridizations with the polarity determinants* smed-notum* [48, 77] and* smed-wnt-1* [47, 78, 79] were performed at different time points during regeneration to further characterize the effects of DPI on polarity. During normal regeneration* smed-notum* follows a distinct pattern of expression. Within 12 HPA,* smed-notum* is expressed at the anterior wound site in a number of distinct cells. By 1 DPA, the number of* smed-notum*-expressing cells decreases and at approximately 3 DPA these* smed-notum*-positive cells gather at the tip of the anterior blastema. In the final phases, between 5 and 8 DPA, the expression of* smed-notum* becomes restricted to a small number of cells located at the tip of the head, along the midline and in the two cephalic ganglia. After DPI exposure,* smed-notum* was normally expressed at the anterior wound sites at the 1 DPA and 3 DPA (Figure 9(a)). At 7 days post amputation* smed-notum* expression seemed to be upregulated in the anterior blastemas after DPI exposure in comparison with the control animals (Figure 9(a)). The accumulation of* smed-notum*-expressing cells in DPI-exposed trunk and tail fragments in comparison with the control animals might be a result of the reduced blastema and mispatterned brain, similar to the expression of* smed-th*,* smed-tbh*, and* smed-ndl-4* (Figure 8). Interestingly,* smed-notum*-expressing cells were also present in some posterior blastemas after DPI exposure at 1 DPA. This defect was no longer visible at 3 DPA or 7 DPA. The ectopic expression of* smed-notum* clearly indicates that ROS signaling is required to obtain a correct posterior identity, which is confirmed by the presence of anteriorly situated neuronal cells (dopaminergic* smed-th*-expressing cells and mechanosensory* smed-cintillo*-expressing cells, Figure 8) in the posterior blastema of DPI-exposed trunk parts. These data correlate with the decreased wnt/*β*-catenin signaling observed by Love and colleagues in DPI- and APO-exposed* Xenopus*, which also indicates defects in the establishment of posterior identity after inhibition of ROS production [7]. These possible defects on posterior identity were supported by the ectopic expression of* smed-sfrp-1* in DPI-exposed fragments at 7 DPA (Figure 9(a)).* smed-sfrp-1*, an anterior marker related to the Wnt signaling pathway, is expressed at the anterior wound site within 3 HPA during normal regeneration and a strong cluster of* smed-sfrp-1*-expressing cells (similar to intact organisms) is observed starting 1 DPA [80]. An increase of* smed-sfrp-1* expression was observed in both blastemas in the DPI-exposed animals (Figure 9(a)). In addition to the data obtained by the* in situ* hybridizations, the gene expression data show that at 4 HPA both* smed-sfrp-1* and* smed-pbx* were significantly downregulated in DPI-exposed head fragments (*pbx*, *p* = 0.016;* sfrp-1, p* = 0.063, Figure 3, supplementary Table 1) [80, 81].

We further investigated the effects of DPI exposure on polarity and patterning by visualizing the expression of posterior markers,* smed-wnt-1*, and* smed-fz-4* (Figure 9(b)). In amputated control animals,* smed-wnt-1* expression becomes upregulated in distinct cells between 12 HPA and 1 DPA at both the anterior and posterior wound sites. By 3 DPA, the expression of this gene becomes strictly regulated at the posterior blastema while the expression of* smed-wnt-1* at the anterior blastema is lost. By 7 DPA* smed-wnt-1* expression is restricted to a small number of cells located at the tip of the tail. Although* smed-wnt-1* expression remained unaffected by the DPI exposure at early time points of regeneration, expressed at both wound sites at 1 DPA and posterior at 3 DPA,* smed-wnt-1* expression increased at 7 DPA in the posterior blastemas of DPI-exposed head and trunk fragments (Figure 9(b)), indicating that there is a problem with the posterior patterning. Similar results were obtained when the expression of* smed-fz-4* was investigated. During normal regeneration, frizzled 4 (*fz-4*) is expressed at the posterior wound site within 12 hours post amputation, inhibiting activation of *β*-catenin and thus promoting posteriorization [47, 80]. In DPI-exposed regenerated head fragments,* smed-fz-4* expression was slightly decreased at the posterior wound site after DPI exposure (Figure 9(b)). The* smed-fz-4* staining was separated into two spots, which could explain the two-tailed phenotype that was observed in a few DPI-exposed heads (Figure 9(b); supplementary Figure 5). In the DPI-exposed trunk fragments, on the other hand,* smed-fz-4* expression was clearly upregulated at the posterior wound site in comparison with the control animals, but no differences were observed in the anterior blastemas, confirming the patterning problems observed by the* smed-wnt-1* expression (Figure 9(b)). Similarly as with the expression of the neuronal genes,* smed-notum* and* smed-sfrp-1*, the seemingly increased expression of both* smed-wnt-1* and* smed-fz-4* at 7 DPA in the DPI-exposed animals might be the result of the failed formation of the blastemas. However, it is clear that an inhibition of ROS production induces patterning defects as observed by the expression defects of the different genes visualized via* in situ* hybridizations as well as issues with polarization that were indicated by the ectopic expression of* smed-th*,* smed-cintillo*, and* smed-notum*.

We can state that inhibition of ROS production affects the regeneration process by disturbing correct patterning and polarization (as indicated by the expression of different anterior and posterior markers) as well as by impairing (neuro)differentiation. Due to the interaction between both processes, it remains a question whether the effects on patterning and polarity are a consequence of impaired differentiation or whether they are caused by reduced ROS levels independently of the differentiation defects. Moreover, we must take into account that the presence of neurons also influences the regeneration process. Many studies, on both invertebrate and vertebrate model systems, have elucidated the importance of neuronal signaling for proper blastema formation [82–87]. So at this point, it remains unclear whether the effect on regeneration observed after inhibition of the ROS production is a direct effect of the diminished ROS levels or an indirect effect caused by impaired neuronal regeneration. Future experiments are required to solve these questions and to characterize the direct downstream targets of ROS signaling that influence the regeneration process.

## 4. Conclusion

Amputation-induced ROS production was identified as an important early signaling trigger of regeneration. We demonstrated for the first time that ROS are produced independently of the orientation of the wound site and that impaired ROS production leads to loss of the regeneration capacity of both anterior and posterior body parts. Underlying these regeneration defaults, we found that diminished ROS levels did not alter stem cell proliferation but resulted in a failure of the stem cells to differentiate into the required tissues. More specifically, reduced ROS levels prevented successful regeneration of the nervous system, resulting in reduced cephalic ganglia and the ectopic formation of neuronal cells. However, we hypothesize that reactive oxygen species play an important role in the modulation of the differentiation process of all tissues, since they affect the differentiation of early neoblast progeny. The patterning process was also affected by inhibition of ROS production, as was characterized by different anterior and posterior markers. Whether incorrect patterning is a direct result of reduced ROS levels or an indirect result of improper neuroregeneration and differentiation must still be clarified.

In conclusion, we can certainly state that initial redox signaling is crucial for correct anterior and posterior regeneration, including regeneration of the central nervous system. Future research is necessary to unravel the underlying redox-controlled mechanisms that affect the differentiation and patterning processes.

## Supplementary Material

Supplementary Figure 1: Quantification of the ROS-induced fluorescence is presented as well as figures showing the lack of autofluorescence and visualization of ROS production in neuronal-like structures.
Supplementary Figure 2: The ratio of mitochondrial DNA versus nuclear DNA is presented.
Supplementary Figure 3: Shows the visualization and quantification of ROS production following BSO exposure.
Supplementary Figure 4: Defaults induced by mao(RNAi) are presented.
Supplementary Figure 5: Shows the two-tailed phenotype that is often after DPI exposure.
Supplementary Table 1: Presents the transcript levels of the genes of interest in response to DPI exposure.
Supplementary Table 2: Provides additional information concerning the qPCR experiment following the MIQE guidelines.

## Abbreviations

APO: Apocynin
alas1: Aminolevulinic acid synthase
BA: Before amputation
*β*-act: Beta-actin
*β*-cat: Beta-catenin
BSA: Bovine serum albumin
BSO: Buthionine sulfoximine
Carboxy-H_2_DCFDA: 5-(and-6)-carboxy-2′,7′-dichlorodihydrofluorescein diacetate
Carboxy-DCF: 5-(and-6)-carboxy-2′,7′-dichlorofluorescein
cat: Catalase
cdc73: Cell division cycle 73
CNS: Central nervous system
co1: Cytochrome oxidase subunit 1
CuZnSOD: Superoxide dismutase copper zinc
cyb: Cytochrome B
cys: Cystatin
DAPI: 4′,6-Diamidino-2-phenylindole
DIG: Digoxigenin
DMSO: Dimethylsulfoxide
DPA: Days post amputation
DPI: Diphenyleneiodonium
DUOX: Dual oxidase
FACS: Fluorescence-activated cell sorting
fz-4: Frizzled 4
gad: Glutamate decarboxylase
gapdh: Glyceraldehyde-3-phosphate dehydrogenase
gm2a: GM2 ganglioside activator
gpas: G protein *α*-subunit
H_2_O_2_: Hydrogen peroxide
HPA: Hours post amputation
mao: Monoamine oxidase
MAPK: Mitogen-activated protein kinase
MS222: Tricaine methanesulfonate
mtDNA: Mitochondrial DNA
mRNA: Messenger RNA
NB21.11e: Neoblast
BCIP: 5-Bromo-4-chloro-3-indolyl-phosphate
NBT: Nitro blue tetrazolium chloride
ndl-4: Nou-darake-like
ndk: Nou-darake
nDNA: Nuclear DNA
NOX: NADPH oxidase
OMA: Oligomycin A
PBS: Sodium perborate
PBST: PBS with Triton-X
pbx: Pre-B-cell leukemia homeobox
pc2: Prohormone convertase 2
pcna: Proliferating cell nuclear antigen
PFA: Paraformaldehyde
pxn-2: Peroxidasin
qPCR: Quantitative polymerase chain reaction
RNAi: RNA interference
ROS: Reactive oxygen species
rpl13: Ribosomal protein L13
SDS: Sodium dodecyl sulphate
sfrp-1: Secreted frizzled-related protein
smedwi-1: Piwi-like gene 1
smedwi-2: Piwi-like gene 2
SSC: Saline-sodium citrate
TEA: Triethanolamine
th: Tyrosine hydroxylase
tbh: Tyramine beta-hydroxylase
tor: Target of rapamycin
wnt: Wingless-type MMTV integration site.

## References

[1] Brieger K., Schiavone S., Miller F. J., Krause K.-H. (2012). Reactive oxygen species: from health to disease. *Swiss Medical Weekly*.

[2] Alfadda A. A., Sallam R. M. (2012). Reactive oxygen species in health and disease. *Journal of Biomedicine and Biotechnology*.

[3] Bedard K., Krause K.-H. (2007). The NOX family of ROS-generating NADPH oxidases: physiology and pathophysiology. *Physiological Reviews*.

[4] Covarrubias L., Hernández-García D., Schnabel D., Salas-Vidal E., Castro-Obregón S. (2008). Function of reactive oxygen species during animal development: passive or active?. *Developmental Biology*.

[5] Hernández-García D., Wood C. D., Castro-Obregón S., Covarrubias L. (2010). Reactive oxygen species: a radical role in development?. *Free Radical Biology and Medicine*.

[6] Gauron C., Rampon C., Bouzaffour M. (2013). Sustained production of ROS triggers compensatory proliferation and is required for regeneration to proceed. *Scientific reports*.

[7] Love N. R., Chen Y., Ishibashi S. (2013). Amputation-induced reactive oxygen species are required for successful *Xenopus* tadpole tail regeneration. *Nature Cell Biology*.

[8] Seifert A. W., Voss S. R. (2013). Revisiting the relationship between regenerative ability and aging. *BMC Biology*.

[9] Brockes J. P., Kumar A. (2008). Comparative aspects of animal regeneration. *Annual Review of Cell and Developmental Biology*.

[10] Labbé R. M., Irimia M., Currie K. W. (2012). A comparative transcriptomic analysis reveals conserved features of stem cell pluripotency in planarians and mammals. *Stem Cells*.

[11] Önal P., Grün D., Adamidi C. (2012). Gene expression of pluripotency determinants is conserved between mammalian and planarian stem cells. *The EMBO Journal*.

[12] Tan T. C. J., Rahman R., Jaber-Hijazi F. (2012). Telomere maintenance and telomerase activity are differentially regulated in asexual and sexual worms. *Proceedings of the National Academy of Sciences of the United States of America*.

[13] Sköld H. N., Obst M. (2011). Potential for clonal animals in longevity and ageing studies. *Biogerontology*.

[14] Oviedo N. J., Beane W. S. (2009). Regeneration: the origin of cancer or a possible cure?. *Seminars in Cell and Developmental Biology*.

[15] Lambeth J. D. (2004). NOX enzymes and the biology of reactive oxygen. *Nature Reviews Immunology*.

[16] de Barros S., Dehez S., Arnaud E. (2013). Aging-related decrease of human ASC angiogenic potential is reversed by hypoxia preconditioning through ROS production. *Molecular Therapy*.

[17] Aboobaker A. A. (2011). Planarian stem cells: a simple paradigm for regeneration. *Trends in Cell Biology*.

[18] Agata K., Umesono Y. (2008). Brain regeneration from pluripotent stem cells in planarian. *Philosophical transactions of the Royal Society of London Series B, Biological sciences*.

[19] Sánchez Alvarado A. (2007). Stem cells and the Planarian Schmidtea mediterranea. *Comptes Rendus—Biologies*.

[20] Cebrià F. (2007). Regenerating the central nervous system: how easy for planarians!. *Development Genes and Evolution*.

[21] Cebrià F. (2008). Organization of the nervous system in the model planarian *Schmidtea mediterranea*: an immunocytochemical study. *Neuroscience Research*.

[22] Fraguas S., Barberán S., Ibarra B., Stöger L., Cebrià F. (2012). Regeneration of neuronal cell types in Schmidtea mediterranea: an immunohistochemical and expression study. *The International Journal of Developmental Biology*.

[23] Iglesias M., Almuedo-Castillo M., Aboobaker A. A., Saló E. (2011). Early planarian brain regeneration is independent of blastema polarity mediated by the Wnt/*β*-catenin pathway. *Developmental Biology*.

[24] Reddien P. W., Alvarado A. S. (2004). Fundamentals of planarian regeneration. *Annual Review of Cell and Developmental Biology*.

[25] Saló E. (2006). The power of regeneration and the stem-cell kingdom: freshwater planarians (Platyhelminthes). *BioEssays*.

[26] Umesono Y., Agata K. (2009). Evolution and regeneration of the planarian central nervous system. *Development Growth and Differentiation*.

[27] Oviedo N. J., Nicolas C. L., Adams D. S., Levin M. (2008). Planarians: a versatile and powerful model system for molecular studies of regeneration, adult stem cell regulation, aging, and behavior. *Cold Spring Harbor Protocols*.

[28] Coyoy A., Olguín-Albuerne M., Martínez-Briseño P., Morán J. (2013). Role of reactive oxygen species and NADPH-oxidase in the development of rat cerebellum. *Neurochemistry International*.

[29] Dickinson B. C., Peltier J., Stone D., Schaffer D. V., Chang C. J. (2011). Nox2 redox signaling maintains essential cell populations in the brain. *Nature Chemical Biology*.

[30] Kennedy K. A. M., Sandiford S. D. E., Skerjanc I. S., Li S. S.-C. (2012). Reactive oxygen species and the neuronal fate. *Cellular and Molecular Life Sciences*.

[31] le Belle J. E., Orozco N. M., Paucar A. A. (2011). Proliferative neural stem cells have high endogenous ROS levels that regulate self-renewal and neurogenesis in a PI3K/Akt-dependant manner. *Cell Stem Cell*.

[32] Rieger S., Sagasti A. (2011). Hydrogen peroxide promotes injury-induced peripheral sensory axon regeneration in the zebrafish skin. *PLoS Biology*.

[33] Yoneyama M., Kawada K., Gotoh Y., Shiba T., Ogita K. (2010). Endogenous reactive oxygen species are essential for proliferation of neural stem/progenitor cells. *Neurochemistry International*.

[34] Min J. Y., Park M. H., Park M. K. (2006). Staurosporin induces neurite outgrowth through ROS generation in HN33 hippocampal cell lines. *Journal of Neural Transmission*.

[35] Suzukawa K., Miura K., Mitsushita J. (2000). Nerve growth factor-induced neuronal differentiation requires generation of Rac1-regulated reactive oxygen species. *The Journal of Biological Chemistry*.

[36] Kennedy K. A. M., Ostrakhovitch E. A., Sandiford S. D. E. (2010). Mammalian numb-interacting protein 1/dual oxidase maturation factor 1 directs neuronal fate in stem cells. *The Journal of Biological Chemistry*.

[37] Gotenstein J. R., Swale R. E., Fukuda T. (2010). The *C. elegans* peroxidasin PXN-2 is essential for embryonic morphogenesis and inhibits adult axon regeneration. *Development*.

[38] Stevens A. S., Pirotte N., Plusquin M. (2015). Toxicity profiles and solvent-toxicant interference in the planarian. *Journal of Applied Toxicology*.

[39] Wind S., Beuerlein K., Eucker T. (2010). Comparative pharmacology of chemically distinct NADPH oxidase inhibitors. *British Journal of Pharmacology*.

[40] Stefanska J., Pawliczak R. (2008). Apocynin: molecular aptitudes. *Mediators of Inflammation*.

[41] Plusquin M., Stevens A.-S., van Belleghem F. (2012). Physiological and molecular characterisation of cadmium stress in *Schmidtea mediterranea*. *International Journal of Developmental Biology*.

[42] Guo T., Peters A. H. F. M., Newmark P. A. (2006). A Bruno-like gene is required for stem cell maintenance in planarians. *Developmental Cell*.

[43] März M., Seebeck F., Bartscherer K. (2013). A pitx transcription factor controls the establishment and maintenance of the serotonergic lineage in planarians. *Development*.

[44] Moritz S., Stöckle F., Ortmeier C. (2012). Heterogeneity of planarian stem cells in the S/G2/M phase. *The International Journal of Developmental Biology*.

[45] González-Estévez C., Arseni V., Thambyrajah R. S., Felix D. A., Aboobaker A. A. (2009). Diverse miRNA spatial expression patterns suggest important roles in homeostasis and regeneration in planarians. *The International Journal of Developmental Biology*.

[46] Umesono Y., Watanabe K., Agata K. (1997). A planarian orthopedia homolog is specifically expressed in the branch region of both the mature and regenerating brain. *Development Growth and Differentiation*.

[47] Gurley K. A., Elliott S. A., Simakov O., Schmidt H. A., Holstein T. W., Alvarado A. S. (2010). Expression of secreted Wnt pathway components reveals unexpected complexity of the planarian amputation response. *Developmental Biology*.

[48] Petersen C. P., Reddien P. W. (2011). Polarized notum activation at wounds inhibits Wnt function to promote planarian head regeneration. *Science*.

[49] King R. S., Newmark P. A. (2013). In situ hybridization protocol for enhanced detection of gene expression in the planarian Schmidtea mediterranea. *BMC Developmental Biology*.

[50] Chomczynski P., Sacchi N. (2006). The single-step method of RNA isolation by acid guanidinium thiocyanate-phenol-chloroform extraction: twenty-something years on. *Nature Protocols*.

[51] Derveaux S., Vandesompele J., Hellemans J. (2010). How to do successful gene expression analysis using real-time PCR. *Methods*.

[52] Plusquin M., DeGheselle O., Cuypers A. (2012). Reference genes for qPCR assays in toxic metal and salinity stress in two flatworm model organisms. *Ecotoxicology*.

[53] Bustin S. A., Benes V., Garson J. A. (2009). The MIQE guidelines: minimum information for publication of quantitative real-time PCR experiments. *Clinical Chemistry*.

[54] Wenger Y., Buzgariu W., Reiter S., Galliot B. (2014). Injury-induced immune responses in Hydra. *Seminars in Immunology*.

[55] Finkel T. (2012). Signal transduction by mitochondrial oxidants. *The Journal of Biological Chemistry*.

[56] Lenaz G. (2012). Mitochondria and reactive oxygen species. Which role in physiology and pathology?. *Advances in Experimental Medicine and Biology*.

[57] Lee H.-C., Wei Y.-H. (2005). Mitochondrial biogenesis and mitochondrial DNA maintenance of mammalian cells under oxidative stress. *The International Journal of Biochemistry & Cell Biology*.

[58] Kobayashi C. I., Suda T. (2012). Regulation of reactive oxygen species in stem cells and cancer stem cells. *Journal of Cellular Physiology*.

[59] Kaludercic N., Mialet-Perez J., Paolocci N., Parini A., di Lisa F. (2014). Monoamine oxidases as sources of oxidants in the heart. *Journal of Molecular and Cellular Cardiology*.

[60] Schwartz T. L. (2013). A neuroscientific update on monoamine oxidase and its inhibitors. *CNS Spectrums*.

[61] Romero B. T., Evans D. J., Aboobaker A. A. (2012). FACS analysis of the planarian stem cell compartment as a tool to understand regenerative mechanisms. *Methods in Molecular Biology*.

[62] Peiris T. H., Weckerle F., Ozamoto E. (2012). TOR signaling regulates planarian stem cells and controls localized and organismal growth. *Journal of Cell Science*.

[63] González-Estévez C., Felix D. A., Smith M. D. (2012). SMG-1 and mTORC1 act antagonistically to regulate response to injury and growth in planarians. *PLoS Genetics*.

[64] Tu K. C., Pearson B. J., Alvarado A. S. (2012). TORC1 is required to balance cell proliferation and cell death in planarians. *Developmental Biology*.

[65] Vriz S., Reiter S., Galliot B. (2014). Cell death: a program to regenerate. *Current Topics in Developmental Biology*.

[66] Tasaki J., Shibata N., Nishimura O. (2011). ERK signaling controls blastema cell differentiation during planarian regeneration. *Development*.

[67] Reddien P. W., Oviedo N. J., Jennings J. R., Jenkin J. C., Alvarado A. S. (2005). SMEDWI-2 is a PIWI-like protein that regulates planarian stem cells. *Science*.

[68] Eisenhoffer G. T., Kang H., Alvarado A. S. (2008). Molecular analysis of stem cells and their descendants during cell turnover and regeneration in the planarian *Schmidtea mediterranea*. *Cell Stem Cell*.

[69] Okamoto K., Takeuchi K., Agata K. (2005). Neural projections in planarian brain revealed by fluorescent dye tracing. *Zoological Science*.

[70] Cebria F., Kobayashi C., Umesono Y. (2002). FGFR-related gene nou-darake restricts brain tissues to the head region of planarians. *Nature*.

[71] Scimone M. L., Kravarik K. M., Lapan S. W., Reddien P. W. (2014). Neoblast specialization in regeneration of the planarian Schmidtea mediterranea. *Stem Cell Reports*.

[72] Nishimura K., Kitamura Y., Umesono Y. (2008). Identification of glutamic acid decarboxylase gene and distribution of GABAergic nervous system in the planarian *Dugesia japonica*. *Neuroscience*.

[73] Nishimura K., Kitamura Y., Inoue T. (2007). Reconstruction of dopaminergic neural network and locomotion function in planarian regenerates. *Developmental Neurobiology*.

[74] Fraguas S., Barberán S., Cebrià F. (2011). EGFR signaling regulates cell proliferation, differentiation and morphogenesis during planarian regeneration and homeostasis. *Developmental Biology*.

[75] Nishimura K., Kitamura Y., Inoue T. (2008). Characterization of tyramine beta-hydroxylase in planarian *Dugesia japonica*: cloning and expression. *Neurochemistry International*.

[76] Rink J. C., Gurley K. A., Elliott S. A., Alvarado A. S. (2009). Planarian Hh signaling regulates regeneration polarity and links Hh pathway evolution to cilia. *Science*.

[77] Adell T., Salò E., Boutos M., Bartscherer K. (2009). Smed-Evi/Wntless is required for *β*-catenin-dependent and -independent processes during planarian regeneration. *Development*.

[78] Petersen C. P., Reddien P. W. (2009). A wound-induced Wnt expression program controls planarian regeneration polarity. *Proceedings of the National Academy of Sciences of the United States of America*.

[79] Yazawa S., Umesono Y., Hayashi T., Tarui H., Agata K. (2009). Planarian hedgehog/patched establishes anterior-posterior polarity by regulating Wnt signaling. *Proceedings of the National Academy of Sciences of the United States of America*.

[80] Gurley K. A., Rink J. C., Alvarado A. S. (2008). Beta-catenin defines head versus tail identity during planarian regeneration and homeostasis. *Science*.

[81] Blassberg R. A., Felix D. A., Tejada-Romero B., Aziz Aboobaker A. (2013). PBX/extradenticle is required to re-establish axial structures and polarity during planarian regeneration. *Development*.

[82] Kumar A., Godwin J. W., Gates P. B., Garza-Garcia A. A., Brockes J. P. (2007). Molecular basis for the nerve dependence of limb regeneration in an adult vertebrate. *Science*.

[83] Kumar A., Brockes J. P. (2012). Nerve dependence in tissue, organ, and appendage regeneration. *Trends in Neurosciences*.

[84] Stocum D. L. (2011). The role of peripheral nerves in urodele limb regeneration. *The European Journal of Neuroscience*.

[85] Fraguas S., Barberán S., Iglesias M., Rodríguez-Esteban G., Cebrià F. (2014). egr-4, a target of EGFR signaling, is required for the formation of the brain primordia and head regeneration in planarians. *Development*.

[86] Oviedo N. J., Morokuma J., Walentek P. (2010). Long-range neural and gap junction protein-mediated cues control polarity during planarian regeneration. *Developmental Biology*.

[87] Cebrià F., Newmark P. A. (2007). Morphogenesis defects are associated with abnormal nervous system regeneration following *roboA* RNAi in planarians. *Development*.

