# Supplementary material for: Reactive Oxygen Species in Planarian Regeneration: An Upstream Necessity for Correct Patterning and Brain Formation
Source: Oxid Med Cell Longev. 2015 Jun 9;2015:392476. doi: 10.1155/2015/392476 (PMC4477255; doi:10.1155/2015/392476)
Supplement: Supplementary file 1 — Supplementary Figure 1: Quantification of the ROS-induced fluorescence is presented as well as figures showing the lack of autofluorescence and visualization of ROS production in neuronal-like structures. Supplementary Figure 2: The ratio of mitochondrial DNA versus nuclear DNA is presented. Supplementary Figure 3: Shows the visualization and quantification of ROS production following BSO exposure. Supplementary Figure 4: Defaults induced by mao(RNAi) are presented. Supplementary Figure 5: Shows the two-tailed phenotype that is often after DPI exposure. Supplementary Table 1: Presents the transcript levels of the genes of interest in response to DPI exposure. Supplementary Table 2: Provides additional information concerning the qPCR experiment following the MIQE guidelines. [file 392476.f1.pdf]

SUPPLEMENTARY DATA

Figures

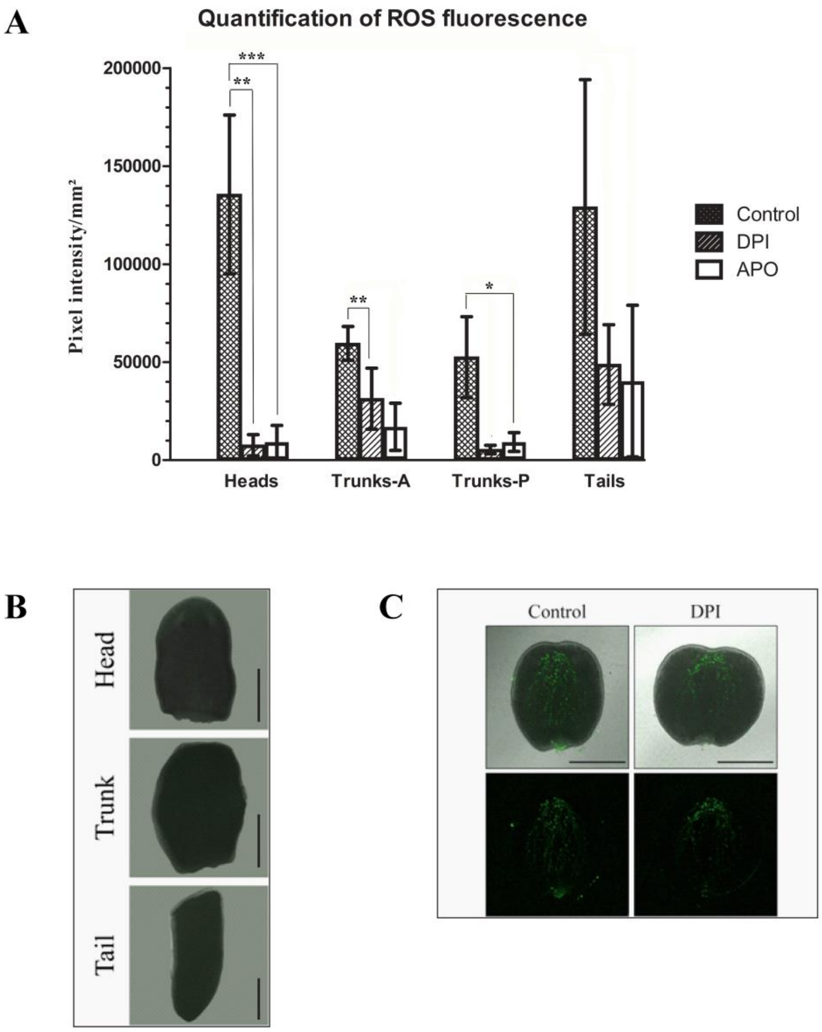

Supplementary figure 1: A) Quantification of ROS fluorescence. The average (± SEM) of the total pixel intensity of the wound site normalized to the total surface area of the worm is given for each wound site and each exposure condition. Statistics were performed on log-transformed data. Significant differences in ROS-induced fluorescence were observed after DPI and APO exposure in the posterior wound sites of the head fragments (control heads: n = 10; DPI-exposed heads: n = 4; APO-exposed heads: n = 2). At the anterior wound site of the trunks, a significant decrease of fluorescence was observed in the DPI-exposed animals (control trunks-A: n = 10; DPI-exposed trunks-A: n = 5; APO-exposed trunks-A: n = 4). At the posterior wound site of the trunks, a significant decrease of fluorescence was observed in the DPI-exposed animals (control trunks-P: n = 11; DPI-exposed trunks-P: n = 4; APO-exposed trunks-P: n = 4). The differences in fluorescence were not significant in the exposed tail fragments in comparison to the controls (control tails: n = 8; DPI-exposed tails: n = 6; APO-exposed tails: n). * $p < 0.1$; ** $p < 0.05$; *** $p < 0.01$. p-values were obtained via one-way ANOVA analyses. B) No autofluorescence was detected at the wound sites after amputation in neither head, trunk or tail fragments (n = 3 for each fragment). C) Observed ROS signaling in neuronal-like structures in regenerating head fragments. Animals were exposed to 3 µM DPI or control medium for at least one hour before the staining procedure. Scale bar A: 500 µm.

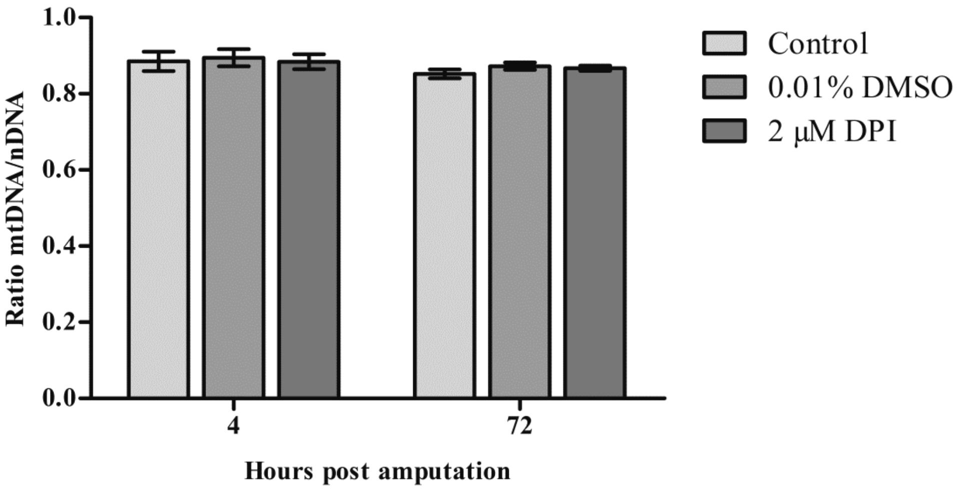

Supplementary figure 2: Ratio mtDNA/nDNA. Regenerating heads (4 HPA and 72 HPA) were exposed to the control medium, 0.01% DMSO or 2 µM DPI. Each bar represents the mean ± SEM of 5 biological replicates. The effect of the treatment was analyzed via a Kruskal-Wallis test, but no significance was observed.

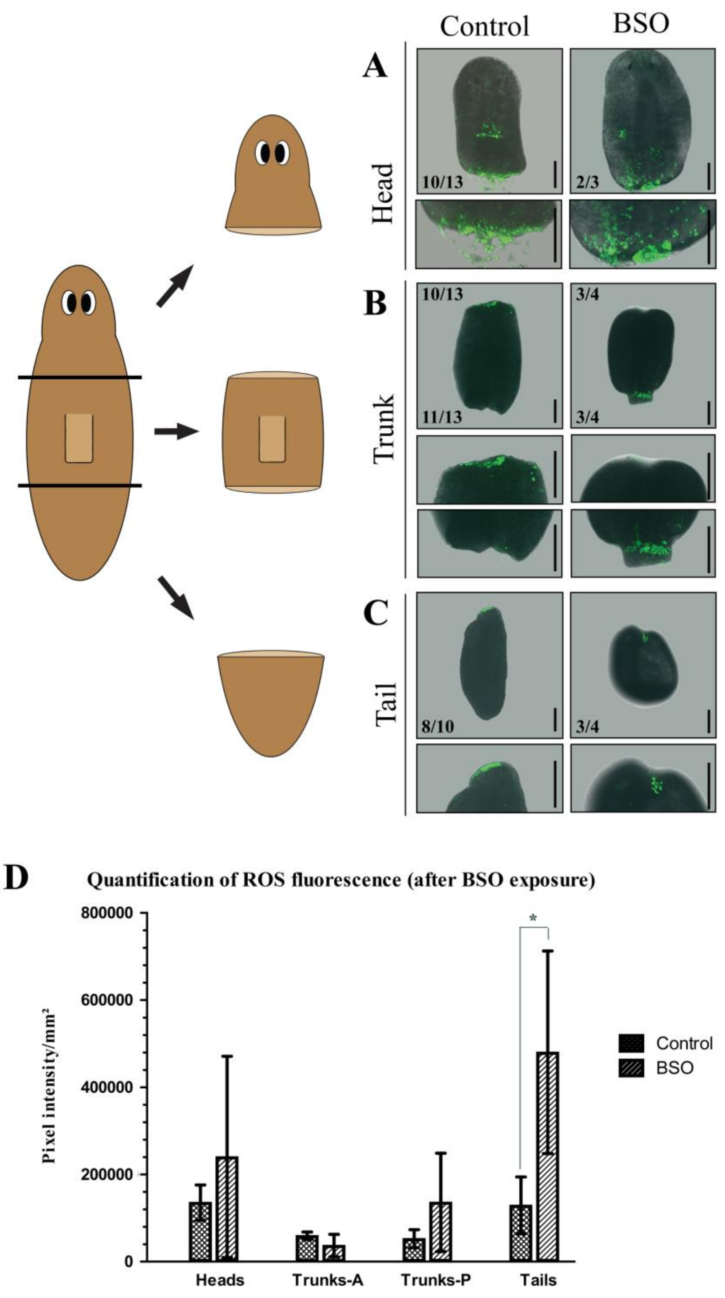

Supplementary figure 3. Visualization of ROS levels using carboxy-H_2DCFDA, 30 minutes after amputation in regenerating head (A), trunk (B) and tail (C) fragments. For each condition an image of the entire animal is shown followed by close-ups of both the anterior and posterior wound sites. ROS are produced at the amputation site in control animals, but ROS levels are visibly increased in BSO-exposed organisms. All animals were exposed for at least one hour before the staining procedure. Scale bars total image: 200 µm, scale bars close-ups: 400 µm. D) Quantification of ROS fluorescence. The average (± SEM) of the total pixel intensity of the wound site normalized to the total surface area of the worm is given for each wound site and each exposure condition. An increased fluorescence is observed after BSO exposure in exposed head, trunk and tail fragments (heads: n = 2; trunks: n = 4; tails: n = 4). A significant difference in ROS-induced fluorescence were observed after BSO exposure in the anterior wound sites of the tail fragments. * $p < 0.1$. p-values were obtained via one-way ANOVA analyses.

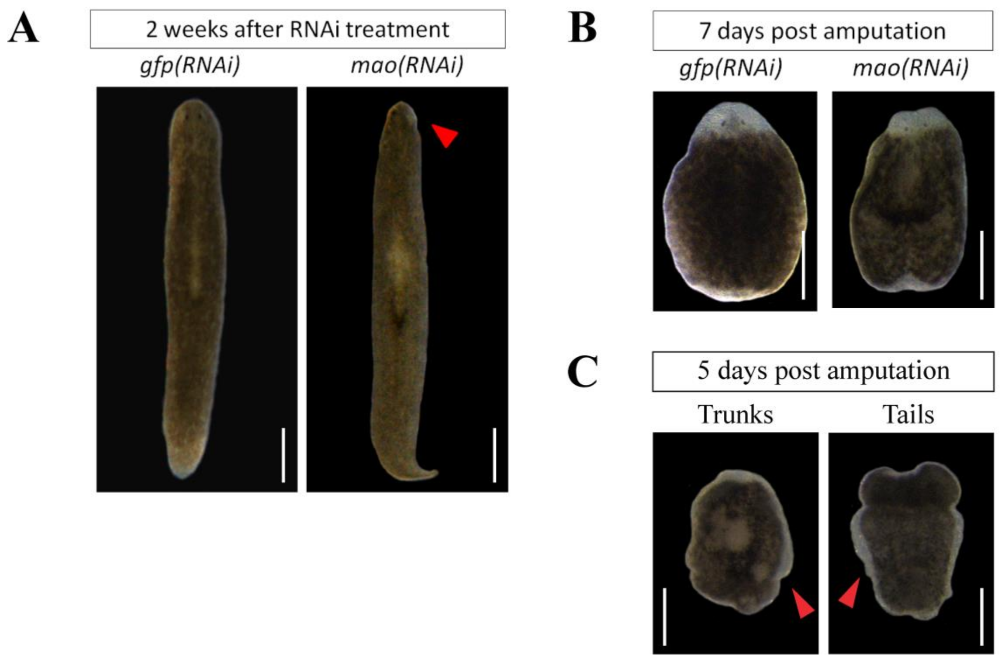

Supplementary figure 4: The effects of *mao(RNAi)* on intact and regenerating planarians. A) Approximately two weeks after the first injections, *moa(RNAi)* animals formed lesion in the head region, as indicated by the red arrow head (n = 5). These animals eventually lost their photoreceptors. Animals were injected for two rounds (2x3 days). Scale bar: 1 mm. B) *mao(RNAi)* animals were able to regenerate proper blastemas and photoreceptors (n = 4). No regeneration defaults were observed at 7 DPA. Regenerating head fragments died within 3 days post amputation. Scale bar: 500 µm. C) Lesions were observed in some of the regenerating *mao(RNAi)* trunk and tail fragments at 5 DPA, as indicated by the red arrow heads. Scale bar: 500 µm.

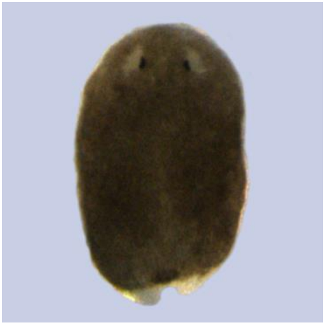

Supplementary figure 5: A two-tail phenotype was occasionally observed in regenerating head fragments after DPI exposure, as can also be observed in figure 5.

Tables

	4 HPA (2 µM DPI)	72 HPA (2 µM DPI)
cat	0.849±0.188	1.163±0.179
CuZnsod	0.950±0.248	1.312±0.035
pbx	0.775±0.066	1.293±0.113
sfrp-1	0.571±0.118	0.975±0.210
fz-4	/	0.916±0.192
smedwi-2	0.777±0.061	1.288±0.044
pcna	0.803±0.246	0.987±0.278
tor	0.677±0.076	1.204±0.171
cdc73	1.026±0.113	0.938±0.055
cyclin-B1	/	1.047±0.183
jnk	0.964±0.061	1.297±0.185
foxo	0.870±0.167	1.161±0.136
pc2	0.525±0.035	1.236±0.183
ndk	0.513±0.058	1.349±0.157
bax	1.052±0.052	1.097±0.076
casp3	0.807±0.124	1.119±0.094
bcl2	1.044±0.337	1.446±0.234

Supplementary table 1. Transcript levels of genes of interest in response to DPI exposure in regenerating head pieces, 4 hours or 72 hours after amputation. Genes of interest include antioxidative genes (*cat, CuZnsod*), genes involved in patterning (*pbx, sfrp-1, fz4*), in differentiation (*smedwi-2, ndk*), cell cycle-related genes (*pcna, tor, cdc73, cyclin-B1, jnk*), neuronal genes (*pc2*) and apoptosis-related genes (*bax, casp3, bcl2*). All transcript values are normalized and expressed relative to the control group (0.01% DMSO) during DPI exposure at 4 hours or 72 hours post amputation. The values indicated in the table are the average ± se of minimum 4 biological replicates. Significant effects (as compared to the corresponding 0.01% DMSO-exposed control worms): ☐ $p < 0.1$; ■ $p < 0.05$. p-values were obtained via one-way ANOVA analyses.

**Experimental design**	
Definition of experimental and control groups	Experimental groups:<br>- Regenerating planarians (head pieces) 3 hours post amputation exposed to 2 µM DPI, dissolved in 0.01% DMSO<br>- Regenerating planarians (head pieces) 72 hours post amputation exposed to 2 µM DPI, dissolved in 0.01% DMSO<br>Control groups:<br>- Regenerating planarians (head pieces) in culture medium<br>- Regenerating planarians (head pieces) in 0.01% DMSO
Number within each group	n ≥ 4
**Sample**	
If frozen, how and how quickly?	Snap frozen in liquid nitrogen
Sample storage conditions and duration	Stored at -70 °C for one month maximally
**Nucleic acid extraction**	
Procedure and/or instrumentation	Frozen animals were disrupted by chemical lysis in 200 µl RNA lysis/binding buffer (Qiagen, Venlo, the Netherlands) including 1% β-mercaptoethanol. RNA was isolated using a phenol-chloroform extraction procedure (14) and was precipitated with Na-acetate and 70% ethanol and resuspended in RNase-free water.
Details of DNase or RNase treatment	Genomic DNA was removed with the Turbo DNA free kit (Ambion).
Nucleid acid quantification	RNA concentrations were assessed using spectrophotometry
Instrument and method	Nanodrop ND-1000 spectrophotometer (NanoDrop Technologies)
Purity	260/280 and 260/230 analysis
**Reverse transcription**	
Complete reaction conditions	SuperScript III First-Strand Synthesis SuperMix for qRT-PCR (Invitrogen)
Amount of RNA and reaction volume	740.4 ng of total RNA in a reaction volume of 20 µl
Storage condition of cDNA	cDNA was 1/10 diluted in water before storage at -20

	°C	
**qPCR target information**		
Gene symbol and sequence accession number	*cat* (mk4.004415.04)[2]; *CuZnsod* (mk4.002589.08)[2]; *pbx* (KC353351.1)[1]; *sfrp-1* (EU130790.1)[1]; *fz-4* (EU130789.1)[1]; *smedwi-2* (DQ186986.1)[1]; *pcna* (mk4.000845.01)[2]; *tor* (JF894291.2)[1]; *cdc73* (FJ588624.1)[1]; *cyclin-B1* (FJ588606.1)[1]; *jnk* (mk4.005863.01)[2]; *foxo* (mk4.016444.00)[2]; *pc2* (BK007043.1)[1]; *ndk* (mk4.003636.00)[2]; *bax* (mk4.009485.00)[2]; *casp3* (mk4.003689.00)[2]; *bcl2* (FJ807655.1)[1]; *cys* (mk4.027397.00)[2]; *β-act* (mk4.000205.04)[2]; *gapdh* (mk4.002051.00)[2]<br><br>([1]NCBI, [2]SMEDGD)	
Amplicon length	*cat* (113); *CuZnsod* (111); *pbx* (101); *sfrp-1* (103); *fz-4* (119); *smedwi-2* (73); *pcna* (114); *tor* (92); *cdc73* (98); *cyclin-B1* (110); *jnk* (92); *foxo* (114); *pc2* (120); *ndk* (109); *bax* (85); *casp3* (91); *bcl-2* (113); *cys* (105); *β-act* (107); *gapdh* (104)	
In silico specificity screen (BLAST)	*cat* (catalase); *CuZnsod* (superoxide dismutase copper zinc); *sfrp-1* (secreted frizzled protein-like protein); *fz-4* (frizzled receptor-like protein); *smedwi-2* (piwi-like protein 2); *pcna* (proliferating cell nuclear antigen); *tor* (target of rapamycin); *cdc73* (cell division cycle 73); *jnk* (c-jun N-terminal kinase); *foxo* (forkhead box O); *pc2* (prohormone convertase 2); *ndk* (nou-darake); *bax* (bcl-2 associated X protein); *casp3* (caspase 3); *bcl-2* (B-cell lymphoma 2); *cys* (cystatin); *β-act* (β-actin); *gapdh* (glyceraldehydes 3-phosphate dehydrogenase)	
Location of each primer by exon or intron	Intron spanning primers: *cat, CuZnsod, foxo, bax, cys*	
**qPCR oligonucleotides**		
Primer sequences	*cat*	5'- CCATTTAGAAATTACGAAGTCGATG-3' (F)<br>5'- AAGTATCTTGGGTTATGTTGAGG- 3' (R)
	CuZnsod	5'- TTCATGCTGTATGCGTTTTG-3' (F)<br>5'-AACCGTGTTTACCAGGAGTTAGA -3' (R)

	pbx	5'- GAGTTATGGCGGTCATTCTG -3' (F)<br>5'- CTGGTTCGTCTTCTCTCATGC -3' (R)
	sfrp-1	5'- CGCTCTGGGGTTGAATCTG -3' (F)<br>5'- GTTGTCGCTGTCGATTTGTG -3' (R)
	fz-4	5'- CGAGGAAAAATCTGCCTGAC -3' (F)<br>5'- CCTTGCATAACGAGCTGGAG -3' (R)
	smedwi-2	5'-GTCATCGTAAAGAAAAGAGTCGGC -3' (F)<br>5'-CCACAACAGTTCCAGGATTTGG -3' (R)
	pcna	5'-TCTTCTCAAGTATCTCTGTCGTTG -3' (F)<br>5'-CTCGTCGTCTTCGATTTTAGG -3' (R)
	tor	5'- GGATTTGTCCAGTCGCATTC -3' (F)<br>5'- CCATCGGGTTGACTCTTAGC -3' (R)
	cdc73	5'- GGAACATATCACGGCAAAGG -3' (F)<br>5'- GGTGCGGGTTGTCTGTTAG -3' (R)
	cyclin-B1	5'- GGTTTCCTACGCGAAAACAG -3' (F)<br>5'- CCGACCAATAGGATCAATGG -3' (R)
	jnk	5'- GTTTCCCGGTACAGATCACA-3' (F)<br>5'-ATGGCTGTAAACGGCTAAGAA-3' (R)
	foxo	5'- CATGAAAAGCTCGTTGTGG-3' (F)<br>5'-TTTAGCAATTTGCGTCTGG-3' (R)
	pc2	5'-ATGACCCTTACCCGTTCC -3' (F)<br>5'-CGTATGCCACTCCAAC-3' (R)
	ndk	5'- ATTCGGCTCAAGAGAAGTGG-3' (F)<br>5'-GAAACACGGAGGTTCATATTTAG-3' (R)
	bax	5'-CAAGTCGGCTTTTAATGATTTCTC -3' (F)<br>5'-AAACAGGTATACGATTGCGTTCCA-3' (R)
	casp3	5'- ATTCAAGCCTGTCGTGGTG-3' (F)<br>5'-CAGCTTCAATTGGAATCTTTTCT-3' (R)
	bcl-2	5'-GGGTCAGAGAAAATGGAGGA -3' (F)<br>5'-TATCCCCAGGGCCACTTT-3' (R)
	cys	5'-AACTCCATGGCTAGAACCGAA -3' (F)<br>5'-CCGTCGGGTAATCCAAGTACA-3' (R)
	β-act	5'- AGAACAGCTTCAGCCTCGTCA-3' (F)<br>5'-TGGAATAGTGCTTCTGGGCAT-3' (R)
	gapd	5'- GCAAAACATTATTCCGGCTTC-3' (F)<br>5'-GCACTGGAACTCTAAAGGCCA-3' (R)
**qPCR protocol**		
Complete reaction conditions	SYBR Green Master Mix (Applied Biosystems)	
Reaction volume and amount of cDNA/DNA	Reaction volume: 10 µl<br>Amount of cDNA: 2.5 µl	
Primer	Primer: 0.3 mM of forward and reverse primer	
Polymerase, Mg2+, dNTP, buffer	Included in the SYBR Green Master Mix (Applied Biosystems)	

Complete thermocycling parameters	Universal cycling conditions:<br>10 min at 95 °C<br>40 cycles: 15 s at 95 °C and 60 s at 60 °C
Manufacturer of qPCR instrument	ABI PRISM 7900 (Applied Biosystems)
**qPCR validation**	
Specificity (gel, sequence, melt or digest)	Samples with a melt temperature (T_m) deviating from the product specific T_m were excluded
For SYBR Green I, C_q of the NTC	NTC of qPCR reaction gave no amplification
PCR efficiency	0.85-1.15
R^2 of calibration curve	≥ 99%
**Data analysis**	
qPCR analysis program (source, version)	qBase (Biogazelle)
Method of C_q determination	SDS software version 2.3 (Applied Biosystems)
Results for NTCs	NTC of qPCR reaction gave no amplification
Justification of number and choice of reference genes	geNorm and Normfinder analyses
Description of normalization method	$2^{-\Delta\Delta Ct}$ (33)
Statistical methods for results significance	Kruskal-Wallis
Software (source, version)	R 3.0.2

Supplementary table 2: MIQE guidelines concerning qPCR experiment (9).
